# Supplementary material for: The association between clinical, subclinical features and autoantibody profile in Vietnamese dermatomyositis patients
Source: PLOS Glob Public Health. 2023 Jan 12;3(1):e0000979. doi: 10.1371/journal.pgph.0000979 (PMC10021228; doi:10.1371/journal.pgph.0000979)
Supplement: S1 File — (PDF) [file pgph.0000979.s002.pdf]

```
 GET
  FILE='C:\Users\ADMIN\Desktop\Datadermatomyositis.sav'.
DATASET NAME DataSet1 WINDOW=FRONT.
RECODE Age (18 thru Highest=2) (ELSE=1) INTO PLage.
EXECUTE.
CROSSTABS
  /TABLES=Gender BY PLage
  /FORMAT=AVALUE TABLES
  /STATISTICS=CHISQ
  /CELLS=COUNT ROW TOTAL
  /COUNT ROUND CELL.
```

Crosstabs

[DataSet1] C:\Users\ADMIN\Desktop\Datadermatomyositis.sav

Case Processing Summary

	Cases					
	Valid		Missing		Total	
	N	Percent	N	Percent	N	Percent
Gender * PLage	72	100.0%	0	0.0%	72	100.0%

Gender * PLage Crosstabulation

			PLage		Total
			JDM	Adult DM	
Gender	female	Count	12	33	45
		% within Gender	26.7%	73.3%	100.0%
		% of Total	16.7%	45.8%	62.5%
	male	Count	5	22	27
		% within Gender	18.5%	81.5%	100.0%
		% of Total	6.9%	30.6%	37.5%
Total		Count	17	55	72
		% within Gender	23.6%	76.4%	100.0%
		% of Total	23.6%	76.4%	100.0%

Chi-Square Tests

	Value	df	Asymp. Sig. (2-sided)	Exact Sig. (2-sided)	Exact Sig. (1-sided)
Pearson Chi-Square	.621[a]	1	.431		
Continuity Correction[b]	.252	1	.616		
Likelihood Ratio	.637	1	.425		
Fisher's Exact Test				.569	.312
Linear-by-Linear Association	.613	1	.434		
N of Valid Cases	72				

a. 0 cells (.0%) have expected count less than 5. The minimum expected count is 6.38.

b. Computed only for a 2x2 table

```
RECODE Age (0 thru 9=1) (9 thru 17=2) (18 thru 40=3) (41 thru 60=4) (61 thru 80=5) (ELSE=6)
EXECUTE.
FREQUENCIES VARIABLES=PLgroupage Age
  /STATISTICS=STDDEV MINIMUM MAXIMUM MEAN MEDIAN
  /ORDER=ANALYSIS.
```

Frequencies

[DataSet1] C:\Users\ADMIN\Desktop\Datadermatomyositis.sav

Statistics

		PLgroupage	Age (year)
N	Valid	72	72
	Missing	0	0
Mean		3.54	41.74
Median		4.00	48.00
Std. Deviation		1.244	22.930
Minimum		1	3
Maximum		6	91

Frequency Table

PLgroupage

		Frequency	Percent	Valid Percent	Cumulative Percent
Valid	0-9 years	4	5.6	5.6	5.6
	10-17 years	13	18.1	18.1	23.6
	18-40 years	15	20.8	20.8	44.4
	41-60 years	21	29.2	29.2	73.6
	61-80 years	18	25.0	25.0	98.6
	over 80 years	1	1.4	1.4	100.0
	Total	72	100.0	100.0	

Age (year)

		Frequency	Percent	Valid Percent	Cumulative Percent
Valid	3	1	1.4	1.4	1.4
	6	2	2.8	2.8	4.2
	8	1	1.4	1.4	5.6
	10	5	6.9	6.9	12.5
	11	2	2.8	2.8	15.3
	13	1	1.4	1.4	16.7
	14	5	6.9	6.9	23.6
	18	2	2.8	2.8	26.4
	19	1	1.4	1.4	27.8
	21	1	1.4	1.4	29.2
	23	1	1.4	1.4	30.6
	24	2	2.8	2.8	33.3
	30	1	1.4	1.4	34.7
	34	1	1.4	1.4	36.1
	35	1	1.4	1.4	37.5
	36	1	1.4	1.4	38.9
	37	2	2.8	2.8	41.7
	38	2	2.8	2.8	44.4
	41	1	1.4	1.4	45.8
	44	1	1.4	1.4	47.2
	46	1	1.4	1.4	48.6
	48	3	4.2	4.2	52.8
	49	1	1.4	1.4	54.2
	50	1	1.4	1.4	55.6
	52	2	2.8	2.8	58.3
	53	3	4.2	4.2	62.5
	54	1	1.4	1.4	63.9
	55	1	1.4	1.4	65.3
	56	1	1.4	1.4	66.7
	57	1	1.4	1.4	68.1
	58	1	1.4	1.4	69.4

Age (year)

	Frequency	Percent	Valid Percent	Cumulative Percent
59	1	1.4	1.4	70.8
60	2	2.8	2.8	73.6
61	2	2.8	2.8	76.4
62	1	1.4	1.4	77.8
63	1	1.4	1.4	79.2
64	2	2.8	2.8	81.9
65	1	1.4	1.4	83.3
66	1	1.4	1.4	84.7
68	2	2.8	2.8	87.5
69	3	4.2	4.2	91.7
70	1	1.4	1.4	93.1
71	1	1.4	1.4	94.4
72	2	2.8	2.8	97.2
74	1	1.4	1.4	98.6
91	1	1.4	1.4	100.0
Total	72	100.0	100.0	

```
 COMPUTE Onsetage=Age - Onset / 12.
EXECUTE.
USE ALL.
COMPUTE filter_$=(PLage = 1).
VARIABLE LABELS filter_$ 'PLage = 1 (FILTER)'.
VALUE LABELS filter_$ 0 'Not Selected' 1 'Selected'.
FORMATS filter_$ (f1.0).
FILTER BY filter_$.
EXECUTE.
FREQUENCIES VARIABLES=Onsetage
  /STATISTICS=STDDEV MINIMUM MAXIMUM MEAN MEDIAN
  /ORDER=ANALYSIS.
```

Frequencies

[DataSet1] C:\Users\ADMIN\Desktop\Datadermatomyositis.sav

Statistics

Onsetage

N	Valid	17
	Missing	0
Mean		8.0049
Median		8.0000
Std. Deviation		3.29410
Minimum		2.83
Maximum		13.83

Onsetage

		Frequency	Percent	Valid Percent	Cumulative Percent
Valid	2.83	1	5.9	5.9	5.9
	3.00	1	5.9	5.9	11.8
	4.83	1	5.9	5.9	17.6
	5.33	1	5.9	5.9	23.5
	5.75	1	5.9	5.9	29.4
	6.00	1	5.9	5.9	35.3
	6.75	1	5.9	5.9	41.2
	7.42	1	5.9	5.9	47.1
	8.00	1	5.9	5.9	52.9
	8.17	1	5.9	5.9	58.8
	8.83	1	5.9	5.9	64.7
	8.83	1	5.9	5.9	70.6
	9.75	1	5.9	5.9	76.5
	11.50	1	5.9	5.9	82.4
	12.25	1	5.9	5.9	88.2
	13.00	1	5.9	5.9	94.1
	13.83	1	5.9	5.9	100.0
	Total	17	100.0	100.0	

```
 USE ALL.
COMPUTE filter_$=(PLage = 2).
VARIABLE LABELS filter_$ 'PLage = 2 (FILTER)'.
VALUE LABELS filter_$ 0 'Not Selected' 1 'Selected'.
FORMATS filter_$ (f1.0).
FILTER BY filter_$.
EXECUTE.
FREQUENCIES VARIABLES=Onsetage
  /STATISTICS=STDDEV MINIMUM MAXIMUM MEAN MEDIAN
  /ORDER=ANALYSIS.
```

Frequencies

[DataSet1] C:\Users\ADMIN\Desktop\Datadermatomyositis.sav

Statistics

Onsetage

N	Valid	55
	Missing	0
Mean		49.2606
Median		51.1667
Std. Deviation		17.68509
Minimum		13.00
Maximum		90.75

Onsetage

		Frequency	Percent	Valid Percent	Cumulative Percent
Valid	13.00	1	1.8	1.8	1.8
	15.58	1	1.8	1.8	3.6
	17.00	1	1.8	1.8	5.5
	18.00	1	1.8	1.8	7.3
	18.75	1	1.8	1.8	9.1
	19.75	1	1.8	1.8	10.9
	20.08	1	1.8	1.8	12.7
	23.83	1	1.8	1.8	14.5
	29.08	1	1.8	1.8	16.4
	30.00	1	1.8	1.8	18.2
	33.17	1	1.8	1.8	20.0
	35.58	1	1.8	1.8	21.8
	36.33	1	1.8	1.8	23.6
	37.58	1	1.8	1.8	25.5
	37.83	1	1.8	1.8	27.3
	39.00	1	1.8	1.8	29.1
	40.42	1	1.8	1.8	30.9
	43.67	1	1.8	1.8	32.7
	44.00	1	1.8	1.8	34.5
	45.92	1	1.8	1.8	36.4
	46.92	1	1.8	1.8	38.2
	47.83	1	1.8	1.8	40.0
	48.58	1	1.8	1.8	41.8
	48.67	1	1.8	1.8	43.6
	49.58	1	1.8	1.8	45.5
	50.33	1	1.8	1.8	47.3
	51.00	1	1.8	1.8	49.1
	51.17	1	1.8	1.8	50.9
	52.00	2	3.6	3.6	54.5
	52.75	1	1.8	1.8	56.4
	54.58	1	1.8	1.8	58.2
	55.08	1	1.8	1.8	60.0

Onsetage

	Frequency	Percent	Valid Percent	Cumulative Percent
58.17	1	1.8	1.8	61.8
59.50	1	1.8	1.8	63.6
59.67	1	1.8	1.8	65.5
60.00	1	1.8	1.8	67.3
60.75	1	1.8	1.8	69.1
60.83	1	1.8	1.8	70.9
61.92	1	1.8	1.8	72.7
62.92	1	1.8	1.8	74.5
63.42	1	1.8	1.8	76.4
63.67	1	1.8	1.8	78.2
65.33	1	1.8	1.8	80.0
65.50	1	1.8	1.8	81.8
66.00	1	1.8	1.8	83.6
66.50	1	1.8	1.8	85.5
66.83	1	1.8	1.8	87.3
67.67	1	1.8	1.8	89.1
67.75	1	1.8	1.8	90.9
67.83	1	1.8	1.8	92.7
70.83	1	1.8	1.8	94.5
71.00	1	1.8	1.8	96.4
73.42	1	1.8	1.8	98.2
90.75	1	1.8	1.8	100.0
Total	55	100.0	100.0	

```
 RECODE Uppergastrointestinaltractsymptoms Lowergastrointestinaltractsymptoms (1=1) (ELSE=0)
EXECUTE.
RECODE Uppergastrointestinaltractsymptoms Lowergastrointestinaltractsymptoms (2=1) (ELSE=0)
EXECUTE.
CROSSTABS
  /TABLES=Fever Fatigue Hairloss PLlossweight Lymphadenopathy Itchy Hypertension Exertionald
  /FORMAT=AVALUE TABLES
  /STATISTICS=CHISQ
  /CELLS=COUNT COLUMN TOTAL
  /COUNT ROUND CELL.
```

```
 CROSSTABS
  /TABLES=Fever Fatigue Hairloss PLlossweight Lymphadenopathy Itchy Hypertension Exertionald
  /FORMAT=AVALUE TABLES
  /STATISTICS=CHISQ
  /CELLS=COUNT COLUMN TOTAL
  /COUNT ROUND CELL.
```

Crosstabs

[DataSet1] C:\Users\ADMIN\Desktop\Datadermatomyositis.sav

Case Processing Summary

	Cases					
	Valid		Missing		Total	
	N	Percent	N	Percent	N	Percent
Fever * PLage	72	100.0%	0	0.0%	72	100.0%
Fatigue * PLage	72	100.0%	0	0.0%	72	100.0%
Hair loss * PLage	72	100.0%	0	0.0%	72	100.0%
PLlossweight * PLage	72	100.0%	0	0.0%	72	100.0%
Lymphadenopathy * PLage	72	100.0%	0	0.0%	72	100.0%
Itchy * PLage	72	100.0%	0	0.0%	72	100.0%
Hypertension * PLage	72	100.0%	0	0.0%	72	100.0%
Exertional dyspnea * PLage	72	100.0%	0	0.0%	72	100.0%
Dry cough * PLage	72	100.0%	0	0.0%	72	100.0%
Dysphagia * PLage	72	100.0%	0	0.0%	72	100.0%
Heartburn * PLage	72	100.0%	0	0.0%	72	100.0%
Diarrhea * PLage	72	100.0%	0	0.0%	72	100.0%
Constipation * PLage	72	100.0%	0	0.0%	72	100.0%
Arthralgia * PLage	72	100.0%	0	0.0%	72	100.0%
Arrhythmia on ECG * PLage	72	100.0%	0	0.0%	72	100.0%

Fever * PLage

Crosstab

			PLage		Total
			JDM	Adult DM	
Fever	No	Count	16	47	63
		% within PLage	94.1%	85.5%	87.5%
		% of Total	22.2%	65.3%	87.5%
	Yes	Count	1	8	9
		% within PLage	5.9%	14.5%	12.5%
		% of Total	1.4%	11.1%	12.5%
Total		Count	17	55	72
		% within PLage	100.0%	100.0%	100.0%
		% of Total	23.6%	76.4%	100.0%

Chi-Square Tests

	Value	df	Asymp. Sig. (2-sided)	Exact Sig. (2-sided)	Exact Sig. (1-sided)
Pearson Chi-Square	.891[a]	1	.345		
Continuity Correction[b]	.275	1	.600		
Likelihood Ratio	1.027	1	.311		
Fisher's Exact Test				.676	.318
N of Valid Cases	72				

a. 1 cells (25.0%) have expected count less than 5. The minimum expected count is 2.13.

b. Computed only for a 2x2 table

Fatigue * PLage

Crosstab

			PLage		Total
			JDM	Adult DM	
Fatigue	No	Count	10	20	30
		% within PLage	58.8%	36.4%	41.7%
		% of Total	13.9%	27.8%	41.7%
	Yes	Count	7	35	42
		% within PLage	41.2%	63.6%	58.3%
		% of Total	9.7%	48.6%	58.3%
Total		Count	17	55	72
		% within PLage	100.0%	100.0%	100.0%
		% of Total	23.6%	76.4%	100.0%

Chi-Square Tests

	Value	df	Asymp. Sig. (2-sided)	Exact Sig. (2-sided)	Exact Sig. (1-sided)
Pearson Chi-Square	2.695[a]	1	.101		
Continuity Correction[b]	1.850	1	.174		
Likelihood Ratio	2.666	1	.103		
Fisher's Exact Test				.158	.087
N of Valid Cases	72				

a. 0 cells (.0%) have expected count less than 5. The minimum expected count is 7.08.

b. Computed only for a 2x2 table

Hair loss * PLage

Crosstab

			PLage		Total
			JDM	Adult DM	
Hair loss	No	Count	14	38	52
		% within PLage	82.4%	69.1%	72.2%
		% of Total	19.4%	52.8%	72.2%
	Yes	Count	3	17	20
		% within PLage	17.6%	30.9%	27.8%
		% of Total	4.2%	23.6%	27.8%
Total		Count	17	55	72
		% within PLage	100.0%	100.0%	100.0%
		% of Total	23.6%	76.4%	100.0%

Chi-Square Tests

	Value	df	Asymp. Sig. (2-sided)	Exact Sig. (2-sided)	Exact Sig. (1-sided)
Pearson Chi-Square	1.138[a]	1	.286		
Continuity Correction[b]	.573	1	.449		
Likelihood Ratio	1.216	1	.270		
Fisher's Exact Test				.364	.228
N of Valid Cases	72				

a. 1 cells (25.0%) have expected count less than 5. The minimum expected count is 4.72.

b. Computed only for a 2x2 table

PLlossweight * PLage

Crosstab

			PLage		Total
			JDM	Adult DM	
PLlossweight	No	Count	13	37	50
		% within PLage	76.5%	67.3%	69.4%
		% of Total	18.1%	51.4%	69.4%
	Yes	Count	4	18	22
		% within PLage	23.5%	32.7%	30.6%
		% of Total	5.6%	25.0%	30.6%
Total		Count	17	55	72
		% within PLage	100.0%	100.0%	100.0%
		% of Total	23.6%	76.4%	100.0%

Chi-Square Tests

	Value	df	Asymp. Sig. (2-sided)	Exact Sig. (2-sided)	Exact Sig. (1-sided)
Pearson Chi-Square	.518[a]	1	.472		
Continuity Correction[b]	.175	1	.676		
Likelihood Ratio	.536	1	.464		
Fisher's Exact Test				.559	.345
Linear-by-Linear Association	.511	1	.475		
N of Valid Cases	72				

a. 0 cells (.0%) have expected count less than 5. The minimum expected count is 5.19.

b. Computed only for a 2x2 table

Lymphadenopathy * PLage

Crosstab

			PLage		Total
			JDM	Adult DM	
Lymphadenopathy	No	Count	14	48	62
		% within PLage	82.4%	87.3%	86.1%
		% of Total	19.4%	66.7%	86.1%
	Yes	Count	3	7	10
		% within PLage	17.6%	12.7%	13.9%
		% of Total	4.2%	9.7%	13.9%
Total		Count	17	55	72
		% within PLage	100.0%	100.0%	100.0%
		% of Total	23.6%	76.4%	100.0%

Chi-Square Tests

	Value	df	Asymp. Sig. (2-sided)	Exact Sig. (2-sided)	Exact Sig. (1-sided)
Pearson Chi-Square	.263[a]	1	.608		
Continuity Correction[b]	.012	1	.911		
Likelihood Ratio	.251	1	.616		
Fisher's Exact Test				.691	.435
N of Valid Cases	72				

a. 1 cells (25.0%) have expected count less than 5. The minimum expected count is 2.36.

b. Computed only for a 2x2 table

Itchy * PLage

Crosstab

			PLage		Total
			JDM	Adult DM	
Itchy	No	Count	16	52	68
		% within PLage	94.1%	94.5%	94.4%
		% of Total	22.2%	72.2%	94.4%
	Yes	Count	1	3	4
		% within PLage	5.9%	5.5%	5.6%
		% of Total	1.4%	4.2%	5.6%
Total		Count	17	55	72
		% within PLage	100.0%	100.0%	100.0%
		% of Total	23.6%	76.4%	100.0%

Chi-Square Tests

	Value	df	Asymp. Sig. (2-sided)	Exact Sig. (2-sided)	Exact Sig. (1-sided)
Pearson Chi-Square	.005[a]	1	.946		
Continuity Correction[b]	.000	1	1.000		
Likelihood Ratio	.004	1	.947		
Fisher's Exact Test				1.000	.668
N of Valid Cases	72				

a. 2 cells (50.0%) have expected count less than 5. The minimum expected count is .94.

b. Computed only for a 2x2 table

Hypertension * PLage

Crosstab

			PLage		Total
			JDM	Adult DM	
Hypertension	No	Count	17	51	68
		% within PLage	100.0%	92.7%	94.4%
		% of Total	23.6%	70.8%	94.4%
	Yes	Count	0	4	4
		% within PLage	0.0%	7.3%	5.6%
		% of Total	0.0%	5.6%	5.6%
Total		Count	17	55	72
		% within PLage	100.0%	100.0%	100.0%
		% of Total	23.6%	76.4%	100.0%

Chi-Square Tests

	Value	df	Asymp. Sig. (2-sided)	Exact Sig. (2-sided)	Exact Sig. (1-sided)
Pearson Chi-Square	1.309[a]	1	.253		
Continuity Correction[b]	.290	1	.590		
Likelihood Ratio	2.226	1	.136		
Fisher's Exact Test				.566	.332
N of Valid Cases	72				

a. 2 cells (50.0%) have expected count less than 5. The minimum expected count is .94.

b. Computed only for a 2x2 table

Exertional dyspnea * PLage

Crosstab

			PLage		Total
			JDM	Adult DM	
Exertional dyspnea	No	Count	16	43	59
		% within PLage	94.1%	78.2%	81.9%
		% of Total	22.2%	59.7%	81.9%
	Yes	Count	1	12	13
		% within PLage	5.9%	21.8%	18.1%
		% of Total	1.4%	16.7%	18.1%
Total		Count	17	55	72
		% within PLage	100.0%	100.0%	100.0%
		% of Total	23.6%	76.4%	100.0%

Chi-Square Tests

	Value	df	Asymp. Sig. (2-sided)	Exact Sig. (2-sided)	Exact Sig. (1-sided)
Pearson Chi-Square	2.229[a]	1	.135		
Continuity Correction[b]	1.282	1	.258		
Likelihood Ratio	2.690	1	.101		
Fisher's Exact Test				.170	.126
N of Valid Cases	72				

a. 1 cells (25.0%) have expected count less than 5. The minimum expected count is 3.07.

b. Computed only for a 2x2 table

Dry cough * PLage

Crosstab

			PLage		Total
			JDM	Adult DM	
Dry cough	No	Count	17	52	69
		% within PLage	100.0%	94.5%	95.8%
		% of Total	23.6%	72.2%	95.8%
	Yes	Count	0	3	3
		% within PLage	0.0%	5.5%	4.2%
		% of Total	0.0%	4.2%	4.2%
Total		Count	17	55	72
		% within PLage	100.0%	100.0%	100.0%
		% of Total	23.6%	76.4%	100.0%

Chi-Square Tests

	Value	df	Asymp. Sig. (2-sided)	Exact Sig. (2-sided)	Exact Sig. (1-sided)
Pearson Chi-Square	.968[a]	1	.325		
Continuity Correction[b]	.084	1	.772		
Likelihood Ratio	1.656	1	.198		
Fisher's Exact Test				1.000	.440
N of Valid Cases	72				

a. 2 cells (50.0%) have expected count less than 5. The minimum expected count is .71.

b. Computed only for a 2x2 table

Dysphagia * PLage

Crosstab

			PLage		Total
			JDM	Adult DM	
Dysphagia	No	Count	16	50	66
		% within PLage	94.1%	90.9%	91.7%
		% of Total	22.2%	69.4%	91.7%
	Yes	Count	1	5	6
		% within PLage	5.9%	9.1%	8.3%
		% of Total	1.4%	6.9%	8.3%
Total		Count	17	55	72
		% within PLage	100.0%	100.0%	100.0%
		% of Total	23.6%	76.4%	100.0%

Chi-Square Tests

	Value	df	Asymp. Sig. (2-sided)	Exact Sig. (2-sided)	Exact Sig. (1-sided)
Pearson Chi-Square	.175[a]	1	.676		
Continuity Correction[b]	.000	1	1.000		
Likelihood Ratio	.188	1	.665		
Fisher's Exact Test				1.000	.564
Linear-by-Linear Association	.173	1	.678		
N of Valid Cases	72				

a. 2 cells (50.0%) have expected count less than 5. The minimum expected count is 1.42.

b. Computed only for a 2x2 table

Heartburn * PLage

Crosstab

			PLage		Total
			JDM	Adult DM	
Heartburn	No	Count	15	45	60
		% within PLage	88.2%	81.8%	83.3%
		% of Total	20.8%	62.5%	83.3%
	Yes	Count	2	10	12
		% within PLage	11.8%	18.2%	16.7%
		% of Total	2.8%	13.9%	16.7%
Total		Count	17	55	72
		% within PLage	100.0%	100.0%	100.0%
		% of Total	23.6%	76.4%	100.0%

Chi-Square Tests

	Value	df	Asymp. Sig. (2-sided)	Exact Sig. (2-sided)	Exact Sig. (1-sided)
Pearson Chi-Square	.385[a]	1	.535		
Continuity Correction[b]	.062	1	.804		
Likelihood Ratio	.410	1	.522		
Fisher's Exact Test				.719	.420
Linear-by-Linear Association	.380	1	.538		
N of Valid Cases	72				

a. 1 cells (25.0%) have expected count less than 5. The minimum expected count is 2.83.

b. Computed only for a 2x2 table

Diarrhea * PLage

Crosstab

			PLage		Total
			JDM	Adult DM	
Diarrhea	No	Count	17	54	71
		% within PLage	100.0%	98.2%	98.6%
		% of Total	23.6%	75.0%	98.6%
	Yes	Count	0	1	1
		% within PLage	0.0%	1.8%	1.4%
		% of Total	0.0%	1.4%	1.4%
Total		Count	17	55	72
		% within PLage	100.0%	100.0%	100.0%
		% of Total	23.6%	76.4%	100.0%

Chi-Square Tests

	Value	df	Asymp. Sig. (2-sided)	Exact Sig. (2-sided)	Exact Sig. (1-sided)
Pearson Chi-Square	.313[a]	1	.576		
Continuity Correction[b]	.000	1	1.000		
Likelihood Ratio	.543	1	.461		
Fisher's Exact Test				1.000	.764
Linear-by-Linear Association	.309	1	.578		
N of Valid Cases	72				

a. 2 cells (50.0%) have expected count less than 5. The minimum expected count is .24.

b. Computed only for a 2x2 table

Constipation * PLage

Crosstab

			PLage		Total
			JDM	Adult DM	
Constipation	No	Count	17	53	70
		% within PLage	100.0%	96.4%	97.2%
		% of Total	23.6%	73.6%	97.2%
	Yes	Count	0	2	2
		% within PLage	0.0%	3.6%	2.8%
		% of Total	0.0%	2.8%	2.8%
Total		Count	17	55	72
		% within PLage	100.0%	100.0%	100.0%
		% of Total	23.6%	76.4%	100.0%

Chi-Square Tests

	Value	df	Asymp. Sig. (2-sided)	Exact Sig. (2-sided)	Exact Sig. (1-sided)
Pearson Chi-Square	.636[a]	1	.425		
Continuity Correction[b]	.000	1	1.000		
Likelihood Ratio	1.095	1	.295		
Fisher's Exact Test				1.000	.581
Linear-by-Linear Association	.627	1	.428		
N of Valid Cases	72				

a. 2 cells (50.0%) have expected count less than 5. The minimum expected count is .47.

b. Computed only for a 2x2 table

Arthralgia * PLage

Crosstab

			PLage		Total
			JDM	Adult DM	
Arthralgia	No	Count	14	38	52
		% within PLage	82.4%	69.1%	72.2%
		% of Total	19.4%	52.8%	72.2%
	Yes	Count	3	17	20
		% within PLage	17.6%	30.9%	27.8%
		% of Total	4.2%	23.6%	27.8%
Total		Count	17	55	72
		% within PLage	100.0%	100.0%	100.0%
		% of Total	23.6%	76.4%	100.0%

Chi-Square Tests

	Value	df	Asymp. Sig. (2-sided)	Exact Sig. (2-sided)	Exact Sig. (1-sided)
Pearson Chi-Square	1.138[a]	1	.286		
Continuity Correction[b]	.573	1	.449		
Likelihood Ratio	1.216	1	.270		
Fisher's Exact Test				.364	.228
N of Valid Cases	72				

a. 1 cells (25.0%) have expected count less than 5. The minimum expected count is 4.72.

b. Computed only for a 2x2 table

Arrhythmia on ECG * PLage

Crosstab

			PLage		Total
			JDM	Adult DM	
Arrhythmia on ECG	No	Count	17	52	69
		% within PLage	100.0%	94.5%	95.8%
		% of Total	23.6%	72.2%	95.8%
	Yes	Count	0	3	3
		% within PLage	0.0%	5.5%	4.2%
		% of Total	0.0%	4.2%	4.2%
Total		Count	17	55	72
		% within PLage	100.0%	100.0%	100.0%
		% of Total	23.6%	76.4%	100.0%

Chi-Square Tests

	Value	df	Asymp. Sig. (2-sided)	Exact Sig. (2-sided)	Exact Sig. (1-sided)
Pearson Chi-Square	.968[a]	1	.325		
Continuity Correction[b]	.084	1	.772		
Likelihood Ratio	1.656	1	.198		
Fisher's Exact Test				1.000	.440
N of Valid Cases	72				

a. 2 cells (50.0%) have expected count less than 5. The minimum expected count is .71.

b. Computed only for a 2x2 table

```
 IF  (Myalgia = 1 | Muscleatrophy = 1 | Muscleweakness = 1) Clinicalmuscledamage=1.
EXECUTE.
RECODE Clinicalmuscledamage (1=1) (ELSE=0) INTO PLClinicalmuscledamge.
EXECUTE.
CROSSTABS
  /TABLES=Heliotroperash Vsign Shawlsign Gottronspapule Gottronssign Poikiloderma Subcutaneo
shands
    Periungualfissures Periungualtelangiectasia Periungualhaemorrhage Raynaud’
sphenomenon Skinulcers Myalgia Muscleweakness
    Muscleatrophy PLClinicalmuscledamge BY PLage
  /FORMAT=AVALUE TABLES
  /STATISTICS=CHISQ
  /CELLS=COUNT COLUMN TOTAL
  /COUNT ROUND CELL.
```

Crosstabs

```
[DataSet1] C:\Users\ADMIN\Desktop\Datadermatomyositis.sav
```

Case Processing Summary

	Cases					
	Valid		Missing		Total	
	N	Percent	N	Percent	N	Percent
Heliotrope rash * PLage	72	100.0%	0	0.0%	72	100.0%
V-sign * PLage	72	100.0%	0	0.0%	72	100.0%
Shawl sign * PLage	72	100.0%	0	0.0%	72	100.0%
Gottron's papule * PLage	72	100.0%	0	0.0%	72	100.0%
Gottron's sign * PLage	72	100.0%	0	0.0%	72	100.0%
Poikiloderma * PLage	72	100.0%	0	0.0%	72	100.0%
Subcutaneous calcification * PLage	72	100.0%	0	0.0%	72	100.0%
Mechanic’s hands * PLage	72	100.0%	0	0.0%	72	100.0%
Periungual fissures * PLage	72	100.0%	0	0.0%	72	100.0%
Periungual telangiectasia * PLage	72	100.0%	0	0.0%	72	100.0%
Periungual haemorrhage * PLage	72	100.0%	0	0.0%	72	100.0%
Raynaud’s phenomenon * PLage	72	100.0%	0	0.0%	72	100.0%
Skin ulcers * PLage	72	100.0%	0	0.0%	72	100.0%
Myalgia * PLage	72	100.0%	0	0.0%	72	100.0%
Muscle weakness * PLage	72	100.0%	0	0.0%	72	100.0%
Muscle atrophy * PLage	72	100.0%	0	0.0%	72	100.0%
PLClinicalmuscledamge * PLage	72	100.0%	0	0.0%	72	100.0%

Heliotrope rash * PLage

Crosstab

			PLage		Total
			JDM	Adult DM	
Heliotrope rash	No	Count	6	21	27
		% within PLage	35.3%	38.2%	37.5%
		% of Total	8.3%	29.2%	37.5%
	Yes	Count	11	34	45
		% within PLage	64.7%	61.8%	62.5%
		% of Total	15.3%	47.2%	62.5%
Total		Count	17	55	72
		% within PLage	100.0%	100.0%	100.0%
		% of Total	23.6%	76.4%	100.0%

Chi-Square Tests

	Value	df	Asymp. Sig. (2-sided)	Exact Sig. (2-sided)	Exact Sig. (1-sided)
Pearson Chi-Square	.046[a]	1	.830		
Continuity Correction[b]	.000	1	1.000		
Likelihood Ratio	.046	1	.829		
Fisher's Exact Test				1.000	.534
N of Valid Cases	72				

a. 0 cells (.0%) have expected count less than 5. The minimum expected count is 6.38.

b. Computed only for a 2x2 table

V-sign * PLage

Crosstab

			PLage		Total
			JDM	Adult DM	
V-sign	No	Count	10	34	44
		% within PLage	58.8%	61.8%	61.1%
		% of Total	13.9%	47.2%	61.1%
	Yes	Count	7	21	28
		% within PLage	41.2%	38.2%	38.9%
		% of Total	9.7%	29.2%	38.9%
Total		Count	17	55	72
		% within PLage	100.0%	100.0%	100.0%
		% of Total	23.6%	76.4%	100.0%

Chi-Square Tests

	Value	df	Asymp. Sig. (2-sided)	Exact Sig. (2-sided)	Exact Sig. (1-sided)
Pearson Chi-Square	.049[a]	1	.825		
Continuity Correction[b]	.000	1	1.000		
Likelihood Ratio	.049	1	.825		
Fisher's Exact Test				1.000	.521
N of Valid Cases	72				

a. 0 cells (.0%) have expected count less than 5. The minimum expected count is 6.61.

b. Computed only for a 2x2 table

Shawl sign * PLage

Crosstab

			PLage		Total
			JDM	Adult DM	
Shawl sign	No	Count	12	16	28
		% within PLage	70.6%	29.1%	38.9%
		% of Total	16.7%	22.2%	38.9%
	Yes	Count	5	39	44
		% within PLage	29.4%	70.9%	61.1%
		% of Total	6.9%	54.2%	61.1%
Total		Count	17	55	72
		% within PLage	100.0%	100.0%	100.0%
		% of Total	23.6%	76.4%	100.0%

Chi-Square Tests

	Value	df	Asymp. Sig. (2-sided)	Exact Sig. (2-sided)	Exact Sig. (1-sided)
Pearson Chi-Square	9.410[a]	1	.002		
Continuity Correction[b]	7.745	1	.005		
Likelihood Ratio	9.305	1	.002		
Fisher's Exact Test				.004	.003
N of Valid Cases	72				

a. 0 cells (.0%) have expected count less than 5. The minimum expected count is 6.61.

b. Computed only for a 2x2 table

Gottron's papule * PLage

Crosstab

			PLage		Total
			JDM	Adult DM	
Gottron's papule	No	Count	7	27	34
		% within PLage	41.2%	49.1%	47.2%
		% of Total	9.7%	37.5%	47.2%
	Yes	Count	10	28	38
		% within PLage	58.8%	50.9%	52.8%
		% of Total	13.9%	38.9%	52.8%
Total		Count	17	55	72
		% within PLage	100.0%	100.0%	100.0%
		% of Total	23.6%	76.4%	100.0%

Chi-Square Tests

	Value	df	Asymp. Sig. (2-sided)	Exact Sig. (2-sided)	Exact Sig. (1-sided)
Pearson Chi-Square	.326[a]	1	.568		
Continuity Correction[b]	.086	1	.769		
Likelihood Ratio	.328	1	.567		
Fisher's Exact Test				.593	.386
N of Valid Cases	72				

a. 0 cells (.0%) have expected count less than 5. The minimum expected count is 8.03.

b. Computed only for a 2x2 table

Gottron's sign * PLage

Crosstab

			PLage		Total
			JDM	Adult DM	
Gottron's sign	No	Count	6	23	29
		% within PLage	35.3%	41.8%	40.3%
		% of Total	8.3%	31.9%	40.3%
	Yes	Count	11	32	43
		% within PLage	64.7%	58.2%	59.7%
		% of Total	15.3%	44.4%	59.7%
Total		Count	17	55	72
		% within PLage	100.0%	100.0%	100.0%
		% of Total	23.6%	76.4%	100.0%

Chi-Square Tests

	Value	df	Asymp. Sig. (2-sided)	Exact Sig. (2-sided)	Exact Sig. (1-sided)
Pearson Chi-Square	.230[a]	1	.632		
Continuity Correction[b]	.039	1	.844		
Likelihood Ratio	.232	1	.630		
Fisher's Exact Test				.779	.426
N of Valid Cases	72				

a. 0 cells (.0%) have expected count less than 5. The minimum expected count is 6.85.

b. Computed only for a 2x2 table

Poikiloderma * PLage

Crosstab

			PLage		Total
			JDM	Adult DM	
Poikiloderma	No	Count	12	41	53
		% within PLage	70.6%	74.5%	73.6%
		% of Total	16.7%	56.9%	73.6%
	Yes	Count	5	14	19
		% within PLage	29.4%	25.5%	26.4%
		% of Total	6.9%	19.4%	26.4%
Total		Count	17	55	72
		% within PLage	100.0%	100.0%	100.0%
		% of Total	23.6%	76.4%	100.0%

Chi-Square Tests

	Value	df	Asymp. Sig. (2-sided)	Exact Sig. (2-sided)	Exact Sig. (1-sided)
Pearson Chi-Square	.105[a]	1	.746		
Continuity Correction[b]	.000	1	.993		
Likelihood Ratio	.103	1	.748		
Fisher's Exact Test				.759	.486
N of Valid Cases	72				

a. 1 cells (25.0%) have expected count less than 5. The minimum expected count is 4.49.

b. Computed only for a 2x2 table

Subcutaneous calcification * PLage

Crosstab

			PLage		Total
			JDM	Adult DM	
Subcutaneous calcification	No	Count	16	53	69
		% within PLage	94.1%	96.4%	95.8%
		% of Total	22.2%	73.6%	95.8%
	Yes	Count	1	2	3
		% within PLage	5.9%	3.6%	4.2%
		% of Total	1.4%	2.8%	4.2%
Total		Count	17	55	72
		% within PLage	100.0%	100.0%	100.0%
		% of Total	23.6%	76.4%	100.0%

Chi-Square Tests

	Value	df	Asymp. Sig. (2-sided)	Exact Sig. (2-sided)	Exact Sig. (1-sided)
Pearson Chi-Square	.164[a]	1	.685		
Continuity Correction[b]	.000	1	1.000		
Likelihood Ratio	.152	1	.697		
Fisher's Exact Test				.560	.560
N of Valid Cases	72				

a. 2 cells (50.0%) have expected count less than 5. The minimum expected count is .71.

b. Computed only for a 2x2 table

Mechanic's hands * PLage

Crosstab

			PLage		Total
			JDM	Adult DM	
Mechanic's hands	No	Count	17	48	65
		% within PLage	100.0%	87.3%	90.3%
		% of Total	23.6%	66.7%	90.3%
	Yes	Count	0	7	7
		% within PLage	0.0%	12.7%	9.7%
		% of Total	0.0%	9.7%	9.7%
Total		Count	17	55	72
		% within PLage	100.0%	100.0%	100.0%
		% of Total	23.6%	76.4%	100.0%

Chi-Square Tests

	Value	df	Asymp. Sig. (2-sided)	Exact Sig. (2-sided)	Exact Sig. (1-sided)
Pearson Chi-Square	2.397[a]	1	.122		
Continuity Correction[b]	1.166	1	.280		
Likelihood Ratio	3.998	1	.046		
Fisher's Exact Test				.187	.138
N of Valid Cases	72				

a. 1 cells (25.0%) have expected count less than 5. The minimum expected count is 1.65.

b. Computed only for a 2x2 table

Periungual fissures * PLage

Crosstab

			PLage		Total
			JDM	Adult DM	
Periungual fissures	No	Count	16	41	57
		% within PLage	94.1%	74.5%	79.2%
		% of Total	22.2%	56.9%	79.2%
	Yes	Count	1	14	15
		% within PLage	5.9%	25.5%	20.8%
		% of Total	1.4%	19.4%	20.8%
Total		Count	17	55	72
		% within PLage	100.0%	100.0%	100.0%
		% of Total	23.6%	76.4%	100.0%

Chi-Square Tests

	Value	df	Asymp. Sig. (2-sided)	Exact Sig. (2-sided)	Exact Sig. (1-sided)
Pearson Chi-Square	3.016[a]	1	.082		
Continuity Correction[b]	1.946	1	.163		
Likelihood Ratio	3.684	1	.055		
Fisher's Exact Test				.100	.074
N of Valid Cases	72				

a. 1 cells (25.0%) have expected count less than 5. The minimum expected count is 3.54.

b. Computed only for a 2x2 table

Periungual telangiectasia * PLage

Crosstab

			PLage		Total
			JDM	Adult DM	
Periungual telangiectasia	No	Count	10	32	42
		% within PLage	58.8%	58.2%	58.3%
		% of Total	13.9%	44.4%	58.3%
	Yes	Count	7	23	30
		% within PLage	41.2%	41.8%	41.7%
		% of Total	9.7%	31.9%	41.7%
Total		Count	17	55	72
		% within PLage	100.0%	100.0%	100.0%
		% of Total	23.6%	76.4%	100.0%

Chi-Square Tests

	Value	df	Asymp. Sig. (2-sided)	Exact Sig. (2-sided)	Exact Sig. (1-sided)
Pearson Chi-Square	.002[a]	1	.963		
Continuity Correction[b]	.000	1	1.000		
Likelihood Ratio	.002	1	.963		
Fisher's Exact Test				1.000	.596
N of Valid Cases	72				

a. 0 cells (.0%) have expected count less than 5. The minimum expected count is 7.08.

b. Computed only for a 2x2 table

Periungual haemorrhage * PLage

Crosstab

			PLage		Total
			JDM	Adult DM	
Periungual haemorrhage	No	Count	17	49	66
		% within PLage	100.0%	89.1%	91.7%
		% of Total	23.6%	68.1%	91.7%
	Yes	Count	0	6	6
		% within PLage	0.0%	10.9%	8.3%
		% of Total	0.0%	8.3%	8.3%
Total		Count	17	55	72
		% within PLage	100.0%	100.0%	100.0%
		% of Total	23.6%	76.4%	100.0%

Chi-Square Tests

	Value	df	Asymp. Sig. (2-sided)	Exact Sig. (2-sided)	Exact Sig. (1-sided)
Pearson Chi-Square	2.023[a]	1	.155		
Continuity Correction[b]	.847	1	.357		
Likelihood Ratio	3.397	1	.065		
Fisher's Exact Test				.325	.186
N of Valid Cases	72				

a. 2 cells (50.0%) have expected count less than 5. The minimum expected count is 1.42.

b. Computed only for a 2x2 table

Raynaud’s phenomenon * PLage

Crosstab

			PLage		Total
			JDM	Adult DM	
Raynaud’s phenomenon	No	Count	14	47	61
		% within PLage	82.4%	85.5%	84.7%
		% of Total	19.4%	65.3%	84.7%
	Yes	Count	3	8	11
		% within PLage	17.6%	14.5%	15.3%
		% of Total	4.2%	11.1%	15.3%
Total		Count	17	55	72
		% within PLage	100.0%	100.0%	100.0%
		% of Total	23.6%	76.4%	100.0%

Chi-Square Tests

	Value	df	Asymp. Sig. (2-sided)	Exact Sig. (2-sided)	Exact Sig. (1-sided)
Pearson Chi-Square	.097[a]	1	.756		
Continuity Correction[b]	.000	1	1.000		
Likelihood Ratio	.094	1	.759		
Fisher's Exact Test				.714	.510
N of Valid Cases	72				

a. 1 cells (25.0%) have expected count less than 5. The minimum expected count is 2.60.

b. Computed only for a 2x2 table

Skin ulcers * PLage

Crosstab

			PLage		Total
			JDM	Adult DM	
Skin ulcers	No	Count	16	50	66
		% within PLage	94.1%	90.9%	91.7%
		% of Total	22.2%	69.4%	91.7%
	Yes	Count	1	5	6
		% within PLage	5.9%	9.1%	8.3%
		% of Total	1.4%	6.9%	8.3%
Total		Count	17	55	72
		% within PLage	100.0%	100.0%	100.0%
		% of Total	23.6%	76.4%	100.0%

Chi-Square Tests

	Value	df	Asymp. Sig. (2-sided)	Exact Sig. (2-sided)	Exact Sig. (1-sided)
Pearson Chi-Square	.175[a]	1	.676		
Continuity Correction[b]	.000	1	1.000		
Likelihood Ratio	.188	1	.665		
Fisher's Exact Test				1.000	.564
N of Valid Cases	72				

a. 2 cells (50.0%) have expected count less than 5. The minimum expected count is 1.42.

b. Computed only for a 2x2 table

Myalgia * PLage

Crosstab

			PLage		Total
			JDM	Adult DM	
Myalgia	No	Count	3	12	15
		% within PLage	17.6%	21.8%	20.8%
		% of Total	4.2%	16.7%	20.8%
	Yes	Count	14	43	57
		% within PLage	82.4%	78.2%	79.2%
		% of Total	19.4%	59.7%	79.2%
Total		Count	17	55	72
		% within PLage	100.0%	100.0%	100.0%
		% of Total	23.6%	76.4%	100.0%

Chi-Square Tests

	Value	df	Asymp. Sig. (2-sided)	Exact Sig. (2-sided)	Exact Sig. (1-sided)
Pearson Chi-Square	.137[a]	1	.711		
Continuity Correction[b]	.001	1	.977		
Likelihood Ratio	.141	1	.707		
Fisher's Exact Test				1.000	.503
Linear-by-Linear Association	.135	1	.713		
N of Valid Cases	72				

a. 1 cells (25.0%) have expected count less than 5. The minimum expected count is 3.54.

b. Computed only for a 2x2 table

Muscle weakness * PLage

Crosstab

			PLage		Total
			JDM	Adult DM	
Muscle weakness	No	Count	7	22	29
		% within PLage	41.2%	40.0%	40.3%
		% of Total	9.7%	30.6%	40.3%
	Yes	Count	10	33	43
		% within PLage	58.8%	60.0%	59.7%
		% of Total	13.9%	45.8%	59.7%
Total		Count	17	55	72
		% within PLage	100.0%	100.0%	100.0%
		% of Total	23.6%	76.4%	100.0%

Chi-Square Tests

	Value	df	Asymp. Sig. (2-sided)	Exact Sig. (2-sided)	Exact Sig. (1-sided)
Pearson Chi-Square	.007[a]	1	.931		
Continuity Correction[b]	.000	1	1.000		
Likelihood Ratio	.007	1	.931		
Fisher's Exact Test				1.000	.574
Linear-by-Linear Association	.007	1	.932		
N of Valid Cases	72				

a. 0 cells (.0%) have expected count less than 5. The minimum expected count is 6.85.

b. Computed only for a 2x2 table

Muscle atrophy * PLage

Crosstab

			PLage		Total
			JDM	Adult DM	
Muscle atrophy	No	Count	17	49	66
		% within PLage	100.0%	89.1%	91.7%
		% of Total	23.6%	68.1%	91.7%
	Yes	Count	0	6	6
		% within PLage	0.0%	10.9%	8.3%
		% of Total	0.0%	8.3%	8.3%
Total		Count	17	55	72
		% within PLage	100.0%	100.0%	100.0%
		% of Total	23.6%	76.4%	100.0%

Chi-Square Tests

	Value	df	Asymp. Sig. (2-sided)	Exact Sig. (2-sided)	Exact Sig. (1-sided)
Pearson Chi-Square	2.023[a]	1	.155		
Continuity Correction[b]	.847	1	.357		
Likelihood Ratio	3.397	1	.065		
Fisher's Exact Test				.325	.186
Linear-by-Linear Association	1.995	1	.158		
N of Valid Cases	72				

a. 2 cells (50.0%) have expected count less than 5. The minimum expected count is 1.42.

b. Computed only for a 2x2 table

PLClinicalmuscledamge * PLage

Crosstab

			PLage		Total
			JDM	Adult DM	
PLClinicalmuscledamge	No	Count	2	8	10
		% within PLage	11.8%	14.5%	13.9%
		% of Total	2.8%	11.1%	13.9%
	Yes	Count	15	47	62
		% within PLage	88.2%	85.5%	86.1%
		% of Total	20.8%	65.3%	86.1%
Total		Count	17	55	72
		% within PLage	100.0%	100.0%	100.0%
		% of Total	23.6%	76.4%	100.0%

Chi-Square Tests

	Value	df	Asymp. Sig. (2-sided)	Exact Sig. (2-sided)	Exact Sig. (1-sided)
Pearson Chi-Square	.084[a]	1	.772		
Continuity Correction[b]	.000	1	1.000		
Likelihood Ratio	.087	1	.768		
Fisher's Exact Test				1.000	.565
Linear-by-Linear Association	.083	1	.774		
N of Valid Cases	72				

a. 1 cells (25.0%) have expected count less than 5. The minimum expected count is 2.36.

b. Computed only for a 2x2 table

```
 RECODE AST ALT (Lowest thru 40=0) (ELSE=1) INTO ElevatedAST ElevatedALT.
EXECUTE.
RECODE CK (Lowest thru 190=0) (ELSE=1) INTO ElevatedCK.
```

```
EXECUTE.
CROSSTABS
  /TABLES=ElevatedCK ElevatedAST ElevatedALT BY PLClinicalmuscledamge
  /FORMAT=AVALUE TABLES
  /STATISTICS=CHISQ
  /CELLS=COUNT COLUMN TOTAL
  /COUNT ROUND CELL.
```

Crosstabs

[DataSet1] C:\Users\ADMIN\Desktop\Datadermatomyositis.sav

Case Processing Summary

	Cases					
	Valid		Missing		Total	
	N	Percent	N	Percent	N	Percent
ElevatedCK * PLClinicalmuscledamge	72	100.0%	0	0.0%	72	100.0%
ElevatedAST * PLClinicalmuscledamge	72	100.0%	0	0.0%	72	100.0%
ElevatedALT * PLClinicalmuscledamge	72	100.0%	0	0.0%	72	100.0%

ElevatedCK * PLClinicalmuscledamge

Crosstab

			PLClinicalmuscledamge		Total
			No	Yes	
ElevatedCK	No	Count	7	37	44
		% within PLClinicalmuscledamge	70.0%	59.7%	61.1%
		% of Total	9.7%	51.4%	61.1%
	Yes	Count	3	25	28
		% within PLClinicalmuscledamge	30.0%	40.3%	38.9%
		% of Total	4.2%	34.7%	38.9%
Total		Count	10	62	72
		% within PLClinicalmuscledamge	100.0%	100.0%	100.0%
		% of Total	13.9%	86.1%	100.0%

Chi-Square Tests

	Value	df	Asymp. Sig. (2-sided)	Exact Sig. (2-sided)	Exact Sig. (1-sided)
Pearson Chi-Square	.386[a]	1	.534		
Continuity Correction[b]	.074	1	.786		
Likelihood Ratio	.398	1	.528		
Fisher's Exact Test				.730	.401
Linear-by-Linear Association	.381	1	.537		
N of Valid Cases	72				

a. 1 cells (25.0%) have expected count less than 5. The minimum expected count is 3.89.

b. Computed only for a 2x2 table

ElevatedAST * PLClinicalmuscledamge

Crosstab

			PLClinicalmuscledamge		Total
			No	Yes	
ElevatedAST	No	Count	6	36	42
		% within PLClinicalmuscledamge	60.0%	58.1%	58.3%
		% of Total	8.3%	50.0%	58.3%
	Yes	Count	4	26	30
		% within PLClinicalmuscledamge	40.0%	41.9%	41.7%
		% of Total	5.6%	36.1%	41.7%
Total		Count	10	62	72
		% within PLClinicalmuscledamge	100.0%	100.0%	100.0%
		% of Total	13.9%	86.1%	100.0%

Chi-Square Tests

	Value	df	Asymp. Sig. (2-sided)	Exact Sig. (2-sided)	Exact Sig. (1-sided)
Pearson Chi-Square	.013[a]	1	.908		
Continuity Correction[b]	.000	1	1.000		
Likelihood Ratio	.013	1	.908		
Fisher's Exact Test				1.000	.596
Linear-by-Linear Association	.013	1	.909		
N of Valid Cases	72				

a. 1 cells (25.0%) have expected count less than 5. The minimum expected count is 4.17.

b. Computed only for a 2x2 table

ElevatedALT * PLClinicalmuscledamge

Crosstab

			PLClinicalmuscledamge		Total
			No	Yes	
ElevatedALT	No	Count	7	40	47
		% within PLClinicalmuscledamge	70.0%	64.5%	65.3%
		% of Total	9.7%	55.6%	65.3%
	Yes	Count	3	22	25
		% within PLClinicalmuscledamge	30.0%	35.5%	34.7%
		% of Total	4.2%	30.6%	34.7%
Total		Count	10	62	72
		% within PLClinicalmuscledamge	100.0%	100.0%	100.0%
		% of Total	13.9%	86.1%	100.0%

Chi-Square Tests

	Value	df	Asymp. Sig. (2-sided)	Exact Sig. (2-sided)	Exact Sig. (1-sided)
Pearson Chi-Square	.114[a]	1	.735		
Continuity Correction[b]	.000	1	1.000		
Likelihood Ratio	.117	1	.733		
Fisher's Exact Test				1.000	.519
Linear-by-Linear Association	.113	1	.737		
N of Valid Cases	72				

a. 1 cells (25.0%) have expected count less than 5. The minimum expected count is 3.47.

b. Computed only for a 2x2 table

```
 USE ALL.
COMPUTE filter_$=(Ro52 >= 0).
VARIABLE LABELS filter_$ 'Ro52 >= 0 (FILTER)'.
VALUE LABELS filter_$ 0 'Not Selected' 1 'Selected'.
FORMATS filter_$ (f1.0).
FILTER BY filter_$.
EXECUTE.
RECODE Jo1 PL7 PL12 EJ OJ TIF1y MDA5 SAE1 NXP2 SRP Ro52 Ku (11 thru Highest=1) (ELSE=0) INTO
EXECUTE.
IF  (Mi2a >= 11 | Mi2b >= 11) Mi2=1.
EXECUTE.
IF  (PLJo1 = 1 | PLPL7 = 1 | PLPL12 = 1 | PLEJ = 1 | PLOJ = 1) Antisynthetase=1.
EXECUTE.
IF  (PM100 >= 11 | PM75 >= 11) PMScl=1.
EXECUTE.
RECODE Mi2 PMScl Antisynthetase (1=1) (ELSE=0) INTO PLMi2 PLPMScl PLantisynthetase.
EXECUTE.
IF  (PLantisynthetase = 1 | PLTIF1y = 1 | PLMi2 = 1 | PLMDA5 = 1 | PLSAE1 = 1 | PLNXP2 = 1 |
```

```
EXECUTE.
IF  (PLRo52 = 1 | PLKu = 1 | PLPMScl = 1) MAAs=1.
EXECUTE.
RECODE MSAs MAAs (1=1) (ELSE=0) INTO PLMSAs PLMAAs.
EXECUTE.
RECODE Antibodynumber (0=1) (ELSE=0) INTO PLnegative.
EXECUTE.
RECODE Antibodynumber (1=1) (ELSE=0) INTO PL1positive.
EXECUTE.
RECODE Antibodynumber (2=1) (ELSE=0) INTO PL2positive.
EXECUTE.
RECODE Antibodynumber (3 thru Highest=1) (ELSE=0) INTO PLatleast3positive.
EXECUTE.
CROSSTABS
  /TABLES=PLnegative PL1positive PL2positive PLatleast3positive PLMSAs PLantisynthetase PLJo
  /FORMAT=AVALUE TABLES
  /STATISTICS=CHISQ
  /CELLS=COUNT COLUMN TOTAL
  /COUNT ROUND CELL.
```

Crosstabs

[DataSet1] C:\Users\ADMIN\Desktop\Datadermatomyositis.sav

Case Processing Summary

	Cases					
	Valid		Missing		Total	
	N	Percent	N	Percent	N	Percent
PLnegative * PLage	63	100.0%	0	0.0%	63	100.0%
PL1positive * PLage	63	100.0%	0	0.0%	63	100.0%
PL2positive * PLage	63	100.0%	0	0.0%	63	100.0%
PLatleast3positive * PLage	63	100.0%	0	0.0%	63	100.0%
PLMSAs * PLage	63	100.0%	0	0.0%	63	100.0%
PLantisynthetase * PLage	63	100.0%	0	0.0%	63	100.0%
PLJo1 * PLage	63	100.0%	0	0.0%	63	100.0%
PLPL7 * PLage	63	100.0%	0	0.0%	63	100.0%
PLPL12 * PLage	63	100.0%	0	0.0%	63	100.0%
PLEJ * PLage	63	100.0%	0	0.0%	63	100.0%
PLOJ * PLage	63	100.0%	0	0.0%	63	100.0%
PLTIF1y * PLage	63	100.0%	0	0.0%	63	100.0%
PLMi2 * PLage	63	100.0%	0	0.0%	63	100.0%
PLMDA5 * PLage	63	100.0%	0	0.0%	63	100.0%
PLSAE1 * PLage	63	100.0%	0	0.0%	63	100.0%
PLNXP2 * PLage	63	100.0%	0	0.0%	63	100.0%
PLSRP * PLage	63	100.0%	0	0.0%	63	100.0%
PLMAAs * PLage	63	100.0%	0	0.0%	63	100.0%
PLRo52 * PLage	63	100.0%	0	0.0%	63	100.0%

Case Processing Summary

	Cases					
	Valid		Missing		Total	
	N	Percent	N	Percent	N	Percent
PLKu * PLage	63	100.0%	0	0.0%	63	100.0%
PLPMScl * PLage	63	100.0%	0	0.0%	63	100.0%

PLnegative * PLage

Crosstab

			PLage		Total
			JDM	Adult DM	
PLnegative	No	Count	10	40	50
		% within PLage	71.4%	81.6%	79.4%
		% of Total	15.9%	63.5%	79.4%
	Yes	Count	4	9	13
		% within PLage	28.6%	18.4%	20.6%
		% of Total	6.3%	14.3%	20.6%
Total		Count	14	49	63
		% within PLage	100.0%	100.0%	100.0%
		% of Total	22.2%	77.8%	100.0%

Chi-Square Tests

	Value	df	Asymp. Sig. (2-sided)	Exact Sig. (2-sided)	Exact Sig. (1-sided)
Pearson Chi-Square	.692[a]	1	.405		
Continuity Correction[b]	.209	1	.647		
Likelihood Ratio	.654	1	.419		
Fisher's Exact Test				.461	.313
Linear-by-Linear Association	.681	1	.409		
N of Valid Cases	63				

a. 1 cells (25.0%) have expected count less than 5. The minimum expected count is 2.89.

b. Computed only for a 2x2 table

PL1positive * PLage

Crosstab

			PLage		Total
			JDM	Adult DM	
PL1positive	No	Count	10	28	38
		% within PLage	71.4%	57.1%	60.3%
		% of Total	15.9%	44.4%	60.3%
	Yes	Count	4	21	25
		% within PLage	28.6%	42.9%	39.7%
		% of Total	6.3%	33.3%	39.7%
Total		Count	14	49	63
		% within PLage	100.0%	100.0%	100.0%
		% of Total	22.2%	77.8%	100.0%

Chi-Square Tests

	Value	df	Asymp. Sig. (2-sided)	Exact Sig. (2-sided)	Exact Sig. (1-sided)
Pearson Chi-Square	.928[a]	1	.335		
Continuity Correction[b]	.428	1	.513		
Likelihood Ratio	.958	1	.328		
Fisher's Exact Test				.374	.260
Linear-by-Linear Association	.914	1	.339		
N of Valid Cases	63				

a. 0 cells (.0%) have expected count less than 5. The minimum expected count is 5.56.

b. Computed only for a 2x2 table

PL2positive * PLage

Crosstab

			PLage		Total
			JDM	Adult DM	
PL2positive	No	Count	11	38	49
		% within PLage	78.6%	77.6%	77.8%
		% of Total	17.5%	60.3%	77.8%
	Yes	Count	3	11	14
		% within PLage	21.4%	22.4%	22.2%
		% of Total	4.8%	17.5%	22.2%
Total		Count	14	49	63
		% within PLage	100.0%	100.0%	100.0%
		% of Total	22.2%	77.8%	100.0%

Chi-Square Tests

	Value	df	Asymp. Sig. (2-sided)	Exact Sig. (2-sided)	Exact Sig. (1-sided)
Pearson Chi-Square	.007[a]	1	.935		
Continuity Correction[b]	.000	1	1.000		
Likelihood Ratio	.007	1	.935		
Fisher's Exact Test				1.000	.625
Linear-by-Linear Association	.006	1	.936		
N of Valid Cases	63				

a. 1 cells (25.0%) have expected count less than 5. The minimum expected count is 3.11.

b. Computed only for a 2x2 table

PLatleast3positive * PLage

Crosstab

			PLage		Total
			JDM	Adult DM	
PLatleast3positive	No	Count	11	41	52
		% within PLage	78.6%	83.7%	82.5%
		% of Total	17.5%	65.1%	82.5%
	Yes	Count	3	8	11
		% within PLage	21.4%	16.3%	17.5%
		% of Total	4.8%	12.7%	17.5%
Total		Count	14	49	63
		% within PLage	100.0%	100.0%	100.0%
		% of Total	22.2%	77.8%	100.0%

Chi-Square Tests

	Value	df	Asymp. Sig. (2-sided)	Exact Sig. (2-sided)	Exact Sig. (1-sided)
Pearson Chi-Square	.197[a]	1	.657		
Continuity Correction[b]	.002	1	.965		
Likelihood Ratio	.189	1	.664		
Fisher's Exact Test				.696	.462
Linear-by-Linear Association	.194	1	.660		
N of Valid Cases	63				

a. 1 cells (25.0%) have expected count less than 5. The minimum expected count is 2.44.

b. Computed only for a 2x2 table

PLMSAs * PLage

Crosstab

			PLage		Total
			JDM	Adult DM	
PLMSAs	No	Count	5	13	18
		% within PLage	35.7%	26.5%	28.6%
		% of Total	7.9%	20.6%	28.6%
	Yes	Count	9	36	45
		% within PLage	64.3%	73.5%	71.4%
		% of Total	14.3%	57.1%	71.4%
Total		Count	14	49	63
		% within PLage	100.0%	100.0%	100.0%
		% of Total	22.2%	77.8%	100.0%

Chi-Square Tests

	Value	df	Asymp. Sig. (2-sided)	Exact Sig. (2-sided)	Exact Sig. (1-sided)
Pearson Chi-Square	.450[a]	1	.502		
Continuity Correction[b]	.113	1	.737		
Likelihood Ratio	.436	1	.509		
Fisher's Exact Test				.517	.360
Linear-by-Linear Association	.443	1	.506		
N of Valid Cases	63				

a. 1 cells (25.0%) have expected count less than 5. The minimum expected count is 4.00.

b. Computed only for a 2x2 table

PLantisynthetase * PLage

Crosstab

			PLage		Total
			JDM	Adult DM	
PLantisynthetase	No	Count	11	41	52
		% within PLage	78.6%	83.7%	82.5%
		% of Total	17.5%	65.1%	82.5%
	Yes	Count	3	8	11
		% within PLage	21.4%	16.3%	17.5%
		% of Total	4.8%	12.7%	17.5%
Total		Count	14	49	63
		% within PLage	100.0%	100.0%	100.0%
		% of Total	22.2%	77.8%	100.0%

Chi-Square Tests

	Value	df	Asymp. Sig. (2-sided)	Exact Sig. (2-sided)	Exact Sig. (1-sided)
Pearson Chi-Square	.197[a]	1	.657		
Continuity Correction[b]	.002	1	.965		
Likelihood Ratio	.189	1	.664		
Fisher's Exact Test				.696	.462
Linear-by-Linear Association	.194	1	.660		
N of Valid Cases	63				

a. 1 cells (25.0%) have expected count less than 5. The minimum expected count is 2.44.

b. Computed only for a 2x2 table

PLJo1 * PLage

Crosstab

			PLage		Total
			JDM	Adult DM	
PLJo1	No	Count	12	46	58
		% within PLage	85.7%	93.9%	92.1%
		% of Total	19.0%	73.0%	92.1%
	Yes	Count	2	3	5
		% within PLage	14.3%	6.1%	7.9%
		% of Total	3.2%	4.8%	7.9%
Total		Count	14	49	63
		% within PLage	100.0%	100.0%	100.0%
		% of Total	22.2%	77.8%	100.0%

Chi-Square Tests

	Value	df	Asymp. Sig. (2-sided)	Exact Sig. (2-sided)	Exact Sig. (1-sided)
Pearson Chi-Square	.993[a]	1	.319		
Continuity Correction[b]	.190	1	.663		
Likelihood Ratio	.874	1	.350		
Fisher's Exact Test				.307	.307
Linear-by-Linear Association	.977	1	.323		
N of Valid Cases	63				

a. 2 cells (50.0%) have expected count less than 5. The minimum expected count is 1.11.

b. Computed only for a 2x2 table

PLPL7 * PLage

Crosstab

			PLage		Total
			JDM	Adult DM	
PLPL7	No	Count	12	47	59
		% within PLage	85.7%	95.9%	93.7%
		% of Total	19.0%	74.6%	93.7%
	Yes	Count	2	2	4
		% within PLage	14.3%	4.1%	6.3%
		% of Total	3.2%	3.2%	6.3%
Total		Count	14	49	63
		% within PLage	100.0%	100.0%	100.0%
		% of Total	22.2%	77.8%	100.0%

Chi-Square Tests

	Value	df	Asymp. Sig. (2-sided)	Exact Sig. (2-sided)	Exact Sig. (1-sided)
Pearson Chi-Square	1.907[a]	1	.167		
Continuity Correction[b]	.577	1	.448		
Likelihood Ratio	1.600	1	.206		
Fisher's Exact Test				.211	.211
Linear-by-Linear Association	1.877	1	.171		
N of Valid Cases	63				

a. 2 cells (50.0%) have expected count less than 5. The minimum expected count is .89.

b. Computed only for a 2x2 table

PLPL12 * PLage

Crosstab

			PLage		Total
			JDM	Adult DM	
PLPL12	No	Count	14	46	60
		% within PLage	100.0%	93.9%	95.2%
		% of Total	22.2%	73.0%	95.2%
	Yes	Count	0	3	3
		% within PLage	0.0%	6.1%	4.8%
		% of Total	0.0%	4.8%	4.8%
Total		Count	14	49	63
		% within PLage	100.0%	100.0%	100.0%
		% of Total	22.2%	77.8%	100.0%

Chi-Square Tests

	Value	df	Asymp. Sig. (2-sided)	Exact Sig. (2-sided)	Exact Sig. (1-sided)
Pearson Chi-Square	.900[a]	1	.343		
Continuity Correction[b]	.056	1	.813		
Likelihood Ratio	1.550	1	.213		
Fisher's Exact Test				1.000	.464
Linear-by-Linear Association	.886	1	.347		
N of Valid Cases	63				

a. 2 cells (50.0%) have expected count less than 5. The minimum expected count is .67.

b. Computed only for a 2x2 table

PLEJ * PLage

Crosstab

			PLage		Total
			JDM	Adult DM	
PLEJ	No	Count	14	48	62
		% within PLage	100.0%	98.0%	98.4%
		% of Total	22.2%	76.2%	98.4%
	Yes	Count	0	1	1
		% within PLage	0.0%	2.0%	1.6%
		% of Total	0.0%	1.6%	1.6%
Total		Count	14	49	63
		% within PLage	100.0%	100.0%	100.0%
		% of Total	22.2%	77.8%	100.0%

Chi-Square Tests

	Value	df	Asymp. Sig. (2-sided)	Exact Sig. (2-sided)	Exact Sig. (1-sided)
Pearson Chi-Square	.290[a]	1	.590		
Continuity Correction[b]	.000	1	1.000		
Likelihood Ratio	.507	1	.476		
Fisher's Exact Test				1.000	.778
Linear-by-Linear Association	.286	1	.593		
N of Valid Cases	63				

a. 2 cells (50.0%) have expected count less than 5. The minimum expected count is .22.

b. Computed only for a 2x2 table

PLOJ * PLage

Crosstab

			PLage		Total
			JDM	Adult DM	
PLOJ	No	Count	14	49	63
		% within PLage	100.0%	100.0%	100.0%
		% of Total	22.2%	77.8%	100.0%
Total		Count	14	49	63
		% within PLage	100.0%	100.0%	100.0%
		% of Total	22.2%	77.8%	100.0%

Chi-Square Tests

	Value
Pearson Chi-Square	.[a]
N of Valid Cases	63

a. No statistics are computed because PLOJ is a constant.

PLTIF1y * PLage

Crosstab

			PLage		Total
			JDM	Adult DM	
PLTIF1y	No	Count	9	36	45
		% within PLage	64.3%	73.5%	71.4%
		% of Total	14.3%	57.1%	71.4%
	Yes	Count	5	13	18
		% within PLage	35.7%	26.5%	28.6%
		% of Total	7.9%	20.6%	28.6%
Total		Count	14	49	63
		% within PLage	100.0%	100.0%	100.0%
		% of Total	22.2%	77.8%	100.0%

Chi-Square Tests

	Value	df	Asymp. Sig. (2-sided)	Exact Sig. (2-sided)	Exact Sig. (1-sided)
Pearson Chi-Square	.450[a]	1	.502		
Continuity Correction[b]	.113	1	.737		
Likelihood Ratio	.436	1	.509		
Fisher's Exact Test				.517	.360
Linear-by-Linear Association	.443	1	.506		
N of Valid Cases	63				

a. 1 cells (25.0%) have expected count less than 5. The minimum expected count is 4.00.

b. Computed only for a 2x2 table

PLMi2 * PLage

Crosstab

			PLage		Total
			JDM	Adult DM	
PLMi2	No	Count	12	42	54
		% within PLage	85.7%	85.7%	85.7%
		% of Total	19.0%	66.7%	85.7%
	Yes	Count	2	7	9
		% within PLage	14.3%	14.3%	14.3%
		% of Total	3.2%	11.1%	14.3%
Total		Count	14	49	63
		% within PLage	100.0%	100.0%	100.0%
		% of Total	22.2%	77.8%	100.0%

Chi-Square Tests

	Value	df	Asymp. Sig. (2-sided)	Exact Sig. (2-sided)	Exact Sig. (1-sided)
Pearson Chi-Square	.000[a]	1	1.000		
Continuity Correction[b]	.000	1	1.000		
Likelihood Ratio	.000	1	1.000		
Fisher's Exact Test				1.000	.646
Linear-by-Linear Association	.000	1	1.000		
N of Valid Cases	63				

a. 1 cells (25.0%) have expected count less than 5. The minimum expected count is 2.00.

b. Computed only for a 2x2 table

PLMDA5 * PLage

Crosstab

			PLage		Total
			JDM	Adult DM	
PLMDA5	No	Count	13	41	54
		% within PLage	92.9%	83.7%	85.7%
		% of Total	20.6%	65.1%	85.7%
	Yes	Count	1	8	9
		% within PLage	7.1%	16.3%	14.3%
		% of Total	1.6%	12.7%	14.3%
Total		Count	14	49	63
		% within PLage	100.0%	100.0%	100.0%
		% of Total	22.2%	77.8%	100.0%

Chi-Square Tests

	Value	df	Asymp. Sig. (2-sided)	Exact Sig. (2-sided)	Exact Sig. (1-sided)
Pearson Chi-Square	.750[a]	1	.386		
Continuity Correction[b]	.188	1	.665		
Likelihood Ratio	.855	1	.355		
Fisher's Exact Test				.670	.354
Linear-by-Linear Association	.738	1	.390		
N of Valid Cases	63				

a. 1 cells (25.0%) have expected count less than 5. The minimum expected count is 2.00.

b. Computed only for a 2x2 table

PLSAE1 * PLage

Crosstab

			PLage		Total
			JDM	Adult DM	
PLSAE1	No	Count	14	44	58
		% within PLage	100.0%	89.8%	92.1%
		% of Total	22.2%	69.8%	92.1%
	Yes	Count	0	5	5
		% within PLage	0.0%	10.2%	7.9%
		% of Total	0.0%	7.9%	7.9%
Total		Count	14	49	63
		% within PLage	100.0%	100.0%	100.0%
		% of Total	22.2%	77.8%	100.0%

Chi-Square Tests

	Value	df	Asymp. Sig. (2-sided)	Exact Sig. (2-sided)	Exact Sig. (1-sided)
Pearson Chi-Square	1.552[a]	1	.213		
Continuity Correction[b]	.469	1	.493		
Likelihood Ratio	2.634	1	.105		
Fisher's Exact Test				.578	.271
Linear-by-Linear Association	1.527	1	.217		
N of Valid Cases	63				

a. 2 cells (50.0%) have expected count less than 5. The minimum expected count is 1.11.

b. Computed only for a 2x2 table

PLNXP2 * PLage

Crosstab

			PLage		Total
			JDM	Adult DM	
PLNXP2	No	Count	11	47	58
		% within PLage	78.6%	95.9%	92.1%
		% of Total	17.5%	74.6%	92.1%
	Yes	Count	3	2	5
		% within PLage	21.4%	4.1%	7.9%
		% of Total	4.8%	3.2%	7.9%
Total		Count	14	49	63
		% within PLage	100.0%	100.0%	100.0%
		% of Total	22.2%	77.8%	100.0%

Chi-Square Tests

	Value	df	Asymp. Sig. (2-sided)	Exact Sig. (2-sided)	Exact Sig. (1-sided)
Pearson Chi-Square	4.484[a]	1	.034		
Continuity Correction[b]	2.425	1	.119		
Likelihood Ratio	3.669	1	.055		
Fisher's Exact Test				.068	.068
Linear-by-Linear Association	4.413	1	.036		
N of Valid Cases	63				

a. 2 cells (50.0%) have expected count less than 5. The minimum expected count is 1.11.

b. Computed only for a 2x2 table

PLSRP * PLage

Crosstab

			PLage		Total
			JDM	Adult DM	
PLSRP	No	Count	12	46	58
		% within PLage	85.7%	93.9%	92.1%
		% of Total	19.0%	73.0%	92.1%
	Yes	Count	2	3	5
		% within PLage	14.3%	6.1%	7.9%
		% of Total	3.2%	4.8%	7.9%
Total		Count	14	49	63
		% within PLage	100.0%	100.0%	100.0%
		% of Total	22.2%	77.8%	100.0%

Chi-Square Tests

	Value	df	Asymp. Sig. (2-sided)	Exact Sig. (2-sided)	Exact Sig. (1-sided)
Pearson Chi-Square	.993[a]	1	.319		
Continuity Correction[b]	.190	1	.663		
Likelihood Ratio	.874	1	.350		
Fisher's Exact Test				.307	.307
Linear-by-Linear Association	.977	1	.323		
N of Valid Cases	63				

a. 2 cells (50.0%) have expected count less than 5. The minimum expected count is 1.11.

b. Computed only for a 2x2 table

PLMAAs * PLage

Crosstab

			PLage		Total
			JDM	Adult DM	
PLMAAs	No	Count	10	30	40
		% within PLage	71.4%	61.2%	63.5%
		% of Total	15.9%	47.6%	63.5%
	Yes	Count	4	19	23
		% within PLage	28.6%	38.8%	36.5%
		% of Total	6.3%	30.2%	36.5%
Total		Count	14	49	63
		% within PLage	100.0%	100.0%	100.0%
		% of Total	22.2%	77.8%	100.0%

Chi-Square Tests

	Value	df	Asymp. Sig. (2-sided)	Exact Sig. (2-sided)	Exact Sig. (1-sided)
Pearson Chi-Square	.489[a]	1	.484		
Continuity Correction[b]	.148	1	.700		
Likelihood Ratio	.502	1	.478		
Fisher's Exact Test				.546	.356
Linear-by-Linear Association	.481	1	.488		
N of Valid Cases	63				

a. 0 cells (.0%) have expected count less than 5. The minimum expected count is 5.11.

b. Computed only for a 2x2 table

PLRo52 * PLage

Crosstab

			PLage		Total
			JDM	Adult DM	
PLRo52	No	Count	13	36	49
		% within PLage	92.9%	73.5%	77.8%
		% of Total	20.6%	57.1%	77.8%
	Yes	Count	1	13	14
		% within PLage	7.1%	26.5%	22.2%
		% of Total	1.6%	20.6%	22.2%
Total		Count	14	49	63
		% within PLage	100.0%	100.0%	100.0%
		% of Total	22.2%	77.8%	100.0%

Chi-Square Tests

	Value	df	Asymp. Sig. (2-sided)	Exact Sig. (2-sided)	Exact Sig. (1-sided)
Pearson Chi-Square	2.368[a]	1	.124		
Continuity Correction[b]	1.379	1	.240		
Likelihood Ratio	2.842	1	.092		
Fisher's Exact Test				.162	.116
Linear-by-Linear Association	2.330	1	.127		
N of Valid Cases	63				

a. 1 cells (25.0%) have expected count less than 5. The minimum expected count is 3.11.

b. Computed only for a 2x2 table

PLKu * PLage

Crosstab

			PLage		Total
			JDM	Adult DM	
PLKu	No	Count	12	43	55
		% within PLage	85.7%	87.8%	87.3%
		% of Total	19.0%	68.3%	87.3%
	Yes	Count	2	6	8
		% within PLage	14.3%	12.2%	12.7%
		% of Total	3.2%	9.5%	12.7%
Total		Count	14	49	63
		% within PLage	100.0%	100.0%	100.0%
		% of Total	22.2%	77.8%	100.0%

Chi-Square Tests

	Value	df	Asymp. Sig. (2-sided)	Exact Sig. (2-sided)	Exact Sig. (1-sided)
Pearson Chi-Square	.041[a]	1	.840		
Continuity Correction[b]	.000	1	1.000		
Likelihood Ratio	.040	1	.842		
Fisher's Exact Test				1.000	.573
Linear-by-Linear Association	.040	1	.841		
N of Valid Cases	63				

a. 1 cells (25.0%) have expected count less than 5. The minimum expected count is 1.78.

b. Computed only for a 2x2 table

PLPMScl * PLage

Crosstab

			PLage		
			JDM	Adult DM	Total
PLPMScl	No	Count	12	46	58
		% within PLage	85.7%	93.9%	92.1%
		% of Total	19.0%	73.0%	92.1%
	Yes	Count	2	3	5
		% within PLage	14.3%	6.1%	7.9%
		% of Total	3.2%	4.8%	7.9%
Total		Count	14	49	63
		% within PLage	100.0%	100.0%	100.0%
		% of Total	22.2%	77.8%	100.0%

Chi-Square Tests

	Value	df	Asymp. Sig. (2-sided)	Exact Sig. (2-sided)	Exact Sig. (1-sided)
Pearson Chi-Square	.993[a]	1	.319		
Continuity Correction[b]	.190	1	.663		
Likelihood Ratio	.874	1	.350		
Fisher's Exact Test				.307	.307
Linear-by-Linear Association	.977	1	.323		
N of Valid Cases	63				

a. 2 cells (50.0%) have expected count less than 5. The minimum expected count is 1.11.

b. Computed only for a 2x2 table

```
 CROSSTABS
  /TABLES=Heliotroperash Vsign Shawlsign Gottronspapule Gottronssign Poikiloderma Subcutaneo
shands
```

```
    Periungualfissures Periungualtelangiectasia Periungualhaemorrhage Raynaud’
sphenomenon Skinulcers Exertionaldyspnea Fever
    Arthralgia PLClinicalmuscledamge ElevatedCK BY PLantisynthetase PLTIF1y PLRo52 PLMi2 PLM
  /FORMAT=AVALUE TABLES
  /STATISTICS=CHISQ
  /CELLS=COUNT COLUMN TOTAL
  /COUNT ROUND CELL.
```

Crosstabs

[DataSet1] C:\Users\ADMIN\Desktop\Datadermatomyositis.sav

Case Processing Summary

	Cases					
	Valid		Missing		Total	
	N	Percent	N	Percent	N	Percent
Heliotrope rash * PLantisynthetase	63	100.0%	0	0.0%	63	100.0%
Heliotrope rash * PLTIF1y	63	100.0%	0	0.0%	63	100.0%
Heliotrope rash * PLRo52	63	100.0%	0	0.0%	63	100.0%
Heliotrope rash * PLMi2	63	100.0%	0	0.0%	63	100.0%
Heliotrope rash * PLMDA5	63	100.0%	0	0.0%	63	100.0%
V-sign * PLantisynthetase	63	100.0%	0	0.0%	63	100.0%
V-sign * PLTIF1y	63	100.0%	0	0.0%	63	100.0%
V-sign * PLRo52	63	100.0%	0	0.0%	63	100.0%
V-sign * PLMi2	63	100.0%	0	0.0%	63	100.0%
V-sign * PLMDA5	63	100.0%	0	0.0%	63	100.0%
Shawl sign * PLantisynthetase	63	100.0%	0	0.0%	63	100.0%
Shawl sign * PLTIF1y	63	100.0%	0	0.0%	63	100.0%
Shawl sign * PLRo52	63	100.0%	0	0.0%	63	100.0%
Shawl sign * PLMi2	63	100.0%	0	0.0%	63	100.0%
Shawl sign * PLMDA5	63	100.0%	0	0.0%	63	100.0%
Gottron's papule * PLantisynthetase	63	100.0%	0	0.0%	63	100.0%
Gottron's papule * PLTIF1y	63	100.0%	0	0.0%	63	100.0%
Gottron's papule * PLRo52	63	100.0%	0	0.0%	63	100.0%
Gottron's papule * PLMi2	63	100.0%	0	0.0%	63	100.0%
Gottron's papule * PLMDA5	63	100.0%	0	0.0%	63	100.0%
Gottron's sign * PLantisynthetase	63	100.0%	0	0.0%	63	100.0%
Gottron's sign * PLTIF1y	63	100.0%	0	0.0%	63	100.0%
Gottron's sign * PLRo52	63	100.0%	0	0.0%	63	100.0%
Gottron's sign * PLMi2	63	100.0%	0	0.0%	63	100.0%

Case Processing Summary

	Cases					
	Valid		Missing		Total	
	N	Percent	N	Percent	N	Percent
Gottron's sign * PLMDA5	63	100.0%	0	0.0%	63	100.0%
Poikiloderma * PLantisynthetase	63	100.0%	0	0.0%	63	100.0%
Poikiloderma * PLTIF1y	63	100.0%	0	0.0%	63	100.0%
Poikiloderma * PLRo52	63	100.0%	0	0.0%	63	100.0%
Poikiloderma * PLMi2	63	100.0%	0	0.0%	63	100.0%
Poikiloderma * PLMDA5	63	100.0%	0	0.0%	63	100.0%
Subcutaneous calcification * PLantisynthetase	63	100.0%	0	0.0%	63	100.0%
Subcutaneous calcification * PLTIF1y	63	100.0%	0	0.0%	63	100.0%
Subcutaneous calcification * PLRo52	63	100.0%	0	0.0%	63	100.0%
Subcutaneous calcification * PLMi2	63	100.0%	0	0.0%	63	100.0%
Subcutaneous calcification * PLMDA5	63	100.0%	0	0.0%	63	100.0%
Mechanic's hands * PLantisynthetase	63	100.0%	0	0.0%	63	100.0%
Mechanic's hands * PLTIF1y	63	100.0%	0	0.0%	63	100.0%
Mechanic's hands * PLRo52	63	100.0%	0	0.0%	63	100.0%
Mechanic's hands * PLMi2	63	100.0%	0	0.0%	63	100.0%
Mechanic's hands * PLMDA5	63	100.0%	0	0.0%	63	100.0%
Periungual fissures * PLantisynthetase	63	100.0%	0	0.0%	63	100.0%
Periungual fissures * PLTIF1y	63	100.0%	0	0.0%	63	100.0%
Periungual fissures * PLRo52	63	100.0%	0	0.0%	63	100.0%
Periungual fissures * PLMi2	63	100.0%	0	0.0%	63	100.0%
Periungual fissures * PLMDA5	63	100.0%	0	0.0%	63	100.0%
Periungual telangiectasia * PLantisynthetase	63	100.0%	0	0.0%	63	100.0%
Periungual telangiectasia * PLTIF1y	63	100.0%	0	0.0%	63	100.0%
Periungual telangiectasia * PLRo52	63	100.0%	0	0.0%	63	100.0%

Case Processing Summary

	Cases					
	Valid		Missing		Total	
	N	Percent	N	Percent	N	Percent
Periungual telangiectasia * PLMi2	63	100.0%	0	0.0%	63	100.0%
Periungual telangiectasia * PLMDA5	63	100.0%	0	0.0%	63	100.0%
Periungual haemorrhage * PLantisynthetase	63	100.0%	0	0.0%	63	100.0%
Periungual haemorrhage * PLTIF1y	63	100.0%	0	0.0%	63	100.0%
Periungual haemorrhage * PLRo52	63	100.0%	0	0.0%	63	100.0%
Periungual haemorrhage * PLMi2	63	100.0%	0	0.0%	63	100.0%
Periungual haemorrhage * PLMDA5	63	100.0%	0	0.0%	63	100.0%
Raynaud’s phenomenon * PLantisynthetase	63	100.0%	0	0.0%	63	100.0%
Raynaud’s phenomenon * PLTIF1y	63	100.0%	0	0.0%	63	100.0%
Raynaud’s phenomenon * PLRo52	63	100.0%	0	0.0%	63	100.0%
Raynaud’s phenomenon * PLMi2	63	100.0%	0	0.0%	63	100.0%
Raynaud’s phenomenon * PLMDA5	63	100.0%	0	0.0%	63	100.0%
Skin ulcers * PLantisynthetase	63	100.0%	0	0.0%	63	100.0%
Skin ulcers * PLTIF1y	63	100.0%	0	0.0%	63	100.0%
Skin ulcers * PLRo52	63	100.0%	0	0.0%	63	100.0%
Skin ulcers * PLMi2	63	100.0%	0	0.0%	63	100.0%
Skin ulcers * PLMDA5	63	100.0%	0	0.0%	63	100.0%
Exertional dyspnea * PLantisynthetase	63	100.0%	0	0.0%	63	100.0%
Exertional dyspnea * PLTIF1y	63	100.0%	0	0.0%	63	100.0%
Exertional dyspnea * PLRo52	63	100.0%	0	0.0%	63	100.0%
Exertional dyspnea * PLMi2	63	100.0%	0	0.0%	63	100.0%
Exertional dyspnea * PLMDA5	63	100.0%	0	0.0%	63	100.0%
Fever * PLantisynthetase	63	100.0%	0	0.0%	63	100.0%
Fever * PLTIF1y	63	100.0%	0	0.0%	63	100.0%
Fever * PLRo52	63	100.0%	0	0.0%	63	100.0%
Fever * PLMi2	63	100.0%	0	0.0%	63	100.0%

Case Processing Summary

	Cases					
	Valid		Missing		Total	
	N	Percent	N	Percent	N	Percent
Fever * PLMDA5	63	100.0%	0	0.0%	63	100.0%
Arthralgia * PLantisynthetase	63	100.0%	0	0.0%	63	100.0%
Arthralgia * PLTIF1y	63	100.0%	0	0.0%	63	100.0%
Arthralgia * PLRo52	63	100.0%	0	0.0%	63	100.0%
Arthralgia * PLMi2	63	100.0%	0	0.0%	63	100.0%
Arthralgia * PLMDA5	63	100.0%	0	0.0%	63	100.0%
PLClinicalmuscledamge * PLantisynthetase	63	100.0%	0	0.0%	63	100.0%
PLClinicalmuscledamge * PLTIF1y	63	100.0%	0	0.0%	63	100.0%
PLClinicalmuscledamge * PLRo52	63	100.0%	0	0.0%	63	100.0%
PLClinicalmuscledamge * PLMi2	63	100.0%	0	0.0%	63	100.0%
PLClinicalmuscledamge * PLMDA5	63	100.0%	0	0.0%	63	100.0%
ElevatedCK * PLantisynthetase	63	100.0%	0	0.0%	63	100.0%
ElevatedCK * PLTIF1y	63	100.0%	0	0.0%	63	100.0%
ElevatedCK * PLRo52	63	100.0%	0	0.0%	63	100.0%
ElevatedCK * PLMi2	63	100.0%	0	0.0%	63	100.0%
ElevatedCK * PLMDA5	63	100.0%	0	0.0%	63	100.0%

Heliotrope rash * PLantisynthetase

Crosstab

			PLantisynthetase		Total
			No	Yes	
Heliotrope rash	No	Count	19	4	23
		% within PLantisynthetase	36.5%	36.4%	36.5%
		% of Total	30.2%	6.3%	36.5%
	Yes	Count	33	7	40
		% within PLantisynthetase	63.5%	63.6%	63.5%
		% of Total	52.4%	11.1%	63.5%
Total		Count	52	11	63
		% within PLantisynthetase	100.0%	100.0%	100.0%
		% of Total	82.5%	17.5%	100.0%

Chi-Square Tests

	Value	df	Asymp. Sig. (2-sided)	Exact Sig. (2-sided)	Exact Sig. (1-sided)
Pearson Chi-Square	.000[a]	1	.991		
Continuity Correction[b]	.000	1	1.000		
Likelihood Ratio	.000	1	.991		
Fisher's Exact Test				1.000	.637
N of Valid Cases	63				

a. 1 cells (25.0%) have expected count less than 5. The minimum expected count is 4.02.

b. Computed only for a 2x2 table

Heliotrope rash * PLTIF1y

Crosstab

			PLTIF1y		Total
			No	Yes	
Heliotrope rash	No	Count	18	5	23
		% within PLTIF1y	40.0%	27.8%	36.5%
		% of Total	28.6%	7.9%	36.5%
	Yes	Count	27	13	40
		% within PLTIF1y	60.0%	72.2%	63.5%
		% of Total	42.9%	20.6%	63.5%
Total		Count	45	18	63
		% within PLTIF1y	100.0%	100.0%	100.0%
		% of Total	71.4%	28.6%	100.0%

Chi-Square Tests

	Value	df	Asymp. Sig. (2-sided)	Exact Sig. (2-sided)	Exact Sig. (1-sided)
Pearson Chi-Square	.829[a]	1	.363		
Continuity Correction[b]	.385	1	.535		
Likelihood Ratio	.851	1	.356		
Fisher's Exact Test				.403	.270
N of Valid Cases	63				

a. 0 cells (.0%) have expected count less than 5. The minimum expected count is 6.57.

b. Computed only for a 2x2 table

Heliotrope rash * PLRo52

Crosstab

			PLRo52		Total
			No	Yes	
Heliotrope rash	No	Count	19	4	23
		% within PLRo52	38.8%	28.6%	36.5%
		% of Total	30.2%	6.3%	36.5%
	Yes	Count	30	10	40
		% within PLRo52	61.2%	71.4%	63.5%
		% of Total	47.6%	15.9%	63.5%
Total		Count	49	14	63
		% within PLRo52	100.0%	100.0%	100.0%
		% of Total	77.8%	22.2%	100.0%

Chi-Square Tests

	Value	df	Asymp. Sig. (2-sided)	Exact Sig. (2-sided)	Exact Sig. (1-sided)
Pearson Chi-Square	.489[a]	1	.484		
Continuity Correction[b]	.148	1	.700		
Likelihood Ratio	.502	1	.478		
Fisher's Exact Test				.546	.356
N of Valid Cases	63				

a. 0 cells (.0%) have expected count less than 5. The minimum expected count is 5.11.

b. Computed only for a 2x2 table

Heliotrope rash * PLMi2

Crosstab

			PLMi2		Total
			No	Yes	
Heliotrope rash	No	Count	21	2	23
		% within PLMi2	38.9%	22.2%	36.5%
		% of Total	33.3%	3.2%	36.5%
	Yes	Count	33	7	40
		% within PLMi2	61.1%	77.8%	63.5%
		% of Total	52.4%	11.1%	63.5%
Total		Count	54	9	63
		% within PLMi2	100.0%	100.0%	100.0%
		% of Total	85.7%	14.3%	100.0%

Chi-Square Tests

	Value	df	Asymp. Sig. (2-sided)	Exact Sig. (2-sided)	Exact Sig. (1-sided)
Pearson Chi-Square	.924[a]	1	.336		
Continuity Correction[b]	.345	1	.557		
Likelihood Ratio	.986	1	.321		
Fisher's Exact Test				.467	.286
N of Valid Cases	63				

a. 1 cells (25.0%) have expected count less than 5. The minimum expected count is 3.29.

b. Computed only for a 2x2 table

Heliotrope rash * PLMDA5

Crosstab

			PLMDA5		Total
			No	Yes	
Heliotrope rash	No	Count	22	1	23
		% within PLMDA5	40.7%	11.1%	36.5%
		% of Total	34.9%	1.6%	36.5%
	Yes	Count	32	8	40
		% within PLMDA5	59.3%	88.9%	63.5%
		% of Total	50.8%	12.7%	63.5%
Total		Count	54	9	63
		% within PLMDA5	100.0%	100.0%	100.0%
		% of Total	85.7%	14.3%	100.0%

Chi-Square Tests

	Value	df	Asymp. Sig. (2-sided)	Exact Sig. (2-sided)	Exact Sig. (1-sided)
Pearson Chi-Square	2.922[a]	1	.087		
Continuity Correction[b]	1.783	1	.182		
Likelihood Ratio	3.416	1	.065		
Fisher's Exact Test				.137	.086
N of Valid Cases	63				

a. 1 cells (25.0%) have expected count less than 5. The minimum expected count is 3.29.

b. Computed only for a 2x2 table

V-sign * PLantisynthetase

Crosstab

			PLantisynthetase		Total
			No	Yes	
V-sign	No	Count	31	7	38
		% within PLantisynthetase	59.6%	63.6%	60.3%
		% of Total	49.2%	11.1%	60.3%
	Yes	Count	21	4	25
		% within PLantisynthetase	40.4%	36.4%	39.7%
		% of Total	33.3%	6.3%	39.7%
Total		Count	52	11	63
		% within PLantisynthetase	100.0%	100.0%	100.0%
		% of Total	82.5%	17.5%	100.0%

Chi-Square Tests

	Value	df	Asymp. Sig. (2-sided)	Exact Sig. (2-sided)	Exact Sig. (1-sided)
Pearson Chi-Square	.061[a]	1	.804		
Continuity Correction[b]	.000	1	1.000		
Likelihood Ratio	.062	1	.804		
Fisher's Exact Test				1.000	.542
N of Valid Cases	63				

a. 1 cells (25.0%) have expected count less than 5. The minimum expected count is 4.37.

b. Computed only for a 2x2 table

V-sign * PLTIF1y

Crosstab

			PLTIF1y		Total
			No	Yes	
V-sign	No	Count	31	7	38
		% within PLTIF1y	68.9%	38.9%	60.3%
		% of Total	49.2%	11.1%	60.3%
	Yes	Count	14	11	25
		% within PLTIF1y	31.1%	61.1%	39.7%
		% of Total	22.2%	17.5%	39.7%
Total		Count	45	18	63
		% within PLTIF1y	100.0%	100.0%	100.0%
		% of Total	71.4%	28.6%	100.0%

Chi-Square Tests

	Value	df	Asymp. Sig. (2-sided)	Exact Sig. (2-sided)	Exact Sig. (1-sided)
Pearson Chi-Square	4.834[a]	1	.028		
Continuity Correction[b]	3.662	1	.056		
Likelihood Ratio	4.779	1	.029		
Fisher's Exact Test				.045	.028
N of Valid Cases	63				

a. 0 cells (.0%) have expected count less than 5. The minimum expected count is 7.14.

b. Computed only for a 2x2 table

V-sign * PLRo52

Crosstab

			PLRo52		Total
			No	Yes	
V-sign	No	Count	28	10	38
		% within PLRo52	57.1%	71.4%	60.3%
		% of Total	44.4%	15.9%	60.3%
	Yes	Count	21	4	25
		% within PLRo52	42.9%	28.6%	39.7%
		% of Total	33.3%	6.3%	39.7%
Total		Count	49	14	63
		% within PLRo52	100.0%	100.0%	100.0%
		% of Total	77.8%	22.2%	100.0%

Chi-Square Tests

	Value	df	Asymp. Sig. (2-sided)	Exact Sig. (2-sided)	Exact Sig. (1-sided)
Pearson Chi-Square	.928[a]	1	.335		
Continuity Correction[b]	.428	1	.513		
Likelihood Ratio	.958	1	.328		
Fisher's Exact Test				.374	.260
N of Valid Cases	63				

a. 0 cells (.0%) have expected count less than 5. The minimum expected count is 5.56.

b. Computed only for a 2x2 table

V-sign * PLMi2

Crosstab

			PLMi2		Total
			No	Yes	
V-sign	No	Count	35	3	38
		% within PLMi2	64.8%	33.3%	60.3%
		% of Total	55.6%	4.8%	60.3%
	Yes	Count	19	6	25
		% within PLMi2	35.2%	66.7%	39.7%
		% of Total	30.2%	9.5%	39.7%
Total		Count	54	9	63
		% within PLMi2	100.0%	100.0%	100.0%
		% of Total	85.7%	14.3%	100.0%

Chi-Square Tests

	Value	df	Asymp. Sig. (2-sided)	Exact Sig. (2-sided)	Exact Sig. (1-sided)
Pearson Chi-Square	3.194[a]	1	.074		
Continuity Correction[b]	2.014	1	.156		
Likelihood Ratio	3.130	1	.077		
Fisher's Exact Test				.138	.079
N of Valid Cases	63				

a. 1 cells (25.0%) have expected count less than 5. The minimum expected count is 3.57.

b. Computed only for a 2x2 table

V-sign * PLMDA5

Crosstab

			PLMDA5		Total
			No	Yes	
V-sign	No	Count	31	7	38
		% within PLMDA5	57.4%	77.8%	60.3%
		% of Total	49.2%	11.1%	60.3%
	Yes	Count	23	2	25
		% within PLMDA5	42.6%	22.2%	39.7%
		% of Total	36.5%	3.2%	39.7%
Total		Count	54	9	63
		% within PLMDA5	100.0%	100.0%	100.0%
		% of Total	85.7%	14.3%	100.0%

Chi-Square Tests

	Value	df	Asymp. Sig. (2-sided)	Exact Sig. (2-sided)	Exact Sig. (1-sided)
Pearson Chi-Square	1.337[a]	1	.247		
Continuity Correction[b]	.622	1	.430		
Likelihood Ratio	1.430	1	.232		
Fisher's Exact Test				.298	.219
N of Valid Cases	63				

a. 1 cells (25.0%) have expected count less than 5. The minimum expected count is 3.57.

b. Computed only for a 2x2 table

Shawl sign * PLantisynthetase

Crosstab

			PLantisynthetase		
			No	Yes	Total
Shawl sign	No	Count	17	4	21
		% within PLantisynthetase	32.7%	36.4%	33.3%
		% of Total	27.0%	6.3%	33.3%
	Yes	Count	35	7	42
		% within PLantisynthetase	67.3%	63.6%	66.7%
		% of Total	55.6%	11.1%	66.7%
Total		Count	52	11	63
		% within PLantisynthetase	100.0%	100.0%	100.0%
		% of Total	82.5%	17.5%	100.0%

Chi-Square Tests

	Value	df	Asymp. Sig. (2-sided)	Exact Sig. (2-sided)	Exact Sig. (1-sided)
Pearson Chi-Square	.055[a]	1	.814		
Continuity Correction[b]	.000	1	1.000		
Likelihood Ratio	.054	1	.815		
Fisher's Exact Test				1.000	.536
N of Valid Cases	63				

a. 1 cells (25.0%) have expected count less than 5. The minimum expected count is 3.67.

b. Computed only for a 2x2 table

Shawl sign * PLTIF1y

Crosstab

			PLTIF1y		Total
			No	Yes	
Shawl sign	No	Count	16	5	21
		% within PLTIF1y	35.6%	27.8%	33.3%
		% of Total	25.4%	7.9%	33.3%
	Yes	Count	29	13	42
		% within PLTIF1y	64.4%	72.2%	66.7%
		% of Total	46.0%	20.6%	66.7%
Total		Count	45	18	63
		% within PLTIF1y	100.0%	100.0%	100.0%
		% of Total	71.4%	28.6%	100.0%

Chi-Square Tests

	Value	df	Asymp. Sig. (2-sided)	Exact Sig. (2-sided)	Exact Sig. (1-sided)
Pearson Chi-Square	.350[a]	1	.554		
Continuity Correction[b]	.088	1	.767		
Likelihood Ratio	.357	1	.550		
Fisher's Exact Test				.768	.389
N of Valid Cases	63				

a. 0 cells (.0%) have expected count less than 5. The minimum expected count is 6.00.

b. Computed only for a 2x2 table

Shawl sign * PLRo52

Crosstab

			PLRo52		Total
			No	Yes	
Shawl sign	No	Count	17	4	21
		% within PLRo52	34.7%	28.6%	33.3%
		% of Total	27.0%	6.3%	33.3%
	Yes	Count	32	10	42
		% within PLRo52	65.3%	71.4%	66.7%
		% of Total	50.8%	15.9%	66.7%
Total		Count	49	14	63
		% within PLRo52	100.0%	100.0%	100.0%
		% of Total	77.8%	22.2%	100.0%

Chi-Square Tests

	Value	df	Asymp. Sig. (2-sided)	Exact Sig. (2-sided)	Exact Sig. (1-sided)
Pearson Chi-Square	.184[a]	1	.668		
Continuity Correction[b]	.011	1	.915		
Likelihood Ratio	.187	1	.665		
Fisher's Exact Test				.757	.466
N of Valid Cases	63				

a. 1 cells (25.0%) have expected count less than 5. The minimum expected count is 4.67.

b. Computed only for a 2x2 table

Shawl sign * PLMi2

Crosstab

			PLMi2		Total
			No	Yes	
Shawl sign	No	Count	18	3	21
		% within PLMi2	33.3%	33.3%	33.3%
		% of Total	28.6%	4.8%	33.3%
	Yes	Count	36	6	42
		% within PLMi2	66.7%	66.7%	66.7%
		% of Total	57.1%	9.5%	66.7%
Total		Count	54	9	63
		% within PLMi2	100.0%	100.0%	100.0%
		% of Total	85.7%	14.3%	100.0%

Chi-Square Tests

	Value	df	Asymp. Sig. (2-sided)	Exact Sig. (2-sided)	Exact Sig. (1-sided)
Pearson Chi-Square	.000[a]	1	1.000		
Continuity Correction[b]	.000	1	1.000		
Likelihood Ratio	.000	1	1.000		
Fisher's Exact Test				1.000	.637
N of Valid Cases	63				

a. 1 cells (25.0%) have expected count less than 5. The minimum expected count is 3.00.

b. Computed only for a 2x2 table

Shawl sign * PLMDA5

Crosstab

			PLMDA5		Total
			No	Yes	
Shawl sign	No	Count	16	5	21
		% within PLMDA5	29.6%	55.6%	33.3%
		% of Total	25.4%	7.9%	33.3%
	Yes	Count	38	4	42
		% within PLMDA5	70.4%	44.4%	66.7%
		% of Total	60.3%	6.3%	66.7%
Total		Count	54	9	63
		% within PLMDA5	100.0%	100.0%	100.0%
		% of Total	85.7%	14.3%	100.0%

Chi-Square Tests

	Value	df	Asymp. Sig. (2-sided)	Exact Sig. (2-sided)	Exact Sig. (1-sided)
Pearson Chi-Square	2.333[a]	1	.127		
Continuity Correction[b]	1.313	1	.252		
Likelihood Ratio	2.205	1	.138		
Fisher's Exact Test				.146	.127
N of Valid Cases	63				

a. 1 cells (25.0%) have expected count less than 5. The minimum expected count is 3.00.

b. Computed only for a 2x2 table

Gottron's papule * PLantisynthetase

Crosstab

			PLantisynthetase		Total
			No	Yes	
Gottron's papule	No	Count	25	5	30
		% within PLantisynthetase	48.1%	45.5%	47.6%
		% of Total	39.7%	7.9%	47.6%
	Yes	Count	27	6	33
		% within PLantisynthetase	51.9%	54.5%	52.4%
		% of Total	42.9%	9.5%	52.4%
Total		Count	52	11	63
		% within PLantisynthetase	100.0%	100.0%	100.0%
		% of Total	82.5%	17.5%	100.0%

Chi-Square Tests

	Value	df	Asymp. Sig. (2-sided)	Exact Sig. (2-sided)	Exact Sig. (1-sided)
Pearson Chi-Square	.025[a]	1	.874		
Continuity Correction[b]	.000	1	1.000		
Likelihood Ratio	.025	1	.874		
Fisher's Exact Test				1.000	.570
N of Valid Cases	63				

a. 0 cells (.0%) have expected count less than 5. The minimum expected count is 5.24.

b. Computed only for a 2x2 table

Gottron's papule * PLTIF1y

Crosstab

			PLTIF1y		Total
			No	Yes	
Gottron's papule	No	Count	21	9	30
		% within PLTIF1y	46.7%	50.0%	47.6%
		% of Total	33.3%	14.3%	47.6%
	Yes	Count	24	9	33
		% within PLTIF1y	53.3%	50.0%	52.4%
		% of Total	38.1%	14.3%	52.4%
Total		Count	45	18	63
		% within PLTIF1y	100.0%	100.0%	100.0%
		% of Total	71.4%	28.6%	100.0%

Chi-Square Tests

	Value	df	Asymp. Sig. (2-sided)	Exact Sig. (2-sided)	Exact Sig. (1-sided)
Pearson Chi-Square	.057[a]	1	.811		
Continuity Correction[b]	.000	1	1.000		
Likelihood Ratio	.057	1	.811		
Fisher's Exact Test				1.000	.515
N of Valid Cases	63				

a. 0 cells (.0%) have expected count less than 5. The minimum expected count is 8.57.

b. Computed only for a 2x2 table

Gottron's papule * PLRo52

Crosstab

			PLRo52		Total
			No	Yes	
Gottron's papule	No	Count	25	5	30
		% within PLRo52	51.0%	35.7%	47.6%
		% of Total	39.7%	7.9%	47.6%
	Yes	Count	24	9	33
		% within PLRo52	49.0%	64.3%	52.4%
		% of Total	38.1%	14.3%	52.4%
Total		Count	49	14	63
		% within PLRo52	100.0%	100.0%	100.0%
		% of Total	77.8%	22.2%	100.0%

Chi-Square Tests

	Value	df	Asymp. Sig. (2-sided)	Exact Sig. (2-sided)	Exact Sig. (1-sided)
Pearson Chi-Square	1.023[a]	1	.312		
Continuity Correction[b]	.501	1	.479		
Likelihood Ratio	1.036	1	.309		
Fisher's Exact Test				.373	.240
N of Valid Cases	63				

a. 0 cells (.0%) have expected count less than 5. The minimum expected count is 6.67.

b. Computed only for a 2x2 table

Gottron's papule * PLMi2

Crosstab

			PLMi2		Total
			No	Yes	
Gottron's papule	No	Count	25	5	30
		% within PLMi2	46.3%	55.6%	47.6%
		% of Total	39.7%	7.9%	47.6%
	Yes	Count	29	4	33
		% within PLMi2	53.7%	44.4%	52.4%
		% of Total	46.0%	6.3%	52.4%
Total		Count	54	9	63
		% within PLMi2	100.0%	100.0%	100.0%
		% of Total	85.7%	14.3%	100.0%

Chi-Square Tests

	Value	df	Asymp. Sig. (2-sided)	Exact Sig. (2-sided)	Exact Sig. (1-sided)
Pearson Chi-Square	.265[a]	1	.607		
Continuity Correction[b]	.024	1	.877		
Likelihood Ratio	.265	1	.607		
Fisher's Exact Test				.725	.437
N of Valid Cases	63				

a. 2 cells (50.0%) have expected count less than 5. The minimum expected count is 4.29.

b. Computed only for a 2x2 table

Gottron's papule * PLMDA5

Crosstab

			PLMDA5		Total
			No	Yes	
Gottron's papule	No	Count	23	7	30
		% within PLMDA5	42.6%	77.8%	47.6%
		% of Total	36.5%	11.1%	47.6%
	Yes	Count	31	2	33
		% within PLMDA5	57.4%	22.2%	52.4%
		% of Total	49.2%	3.2%	52.4%
Total		Count	54	9	63
		% within PLMDA5	100.0%	100.0%	100.0%
		% of Total	85.7%	14.3%	100.0%

Chi-Square Tests

	Value	df	Asymp. Sig. (2-sided)	Exact Sig. (2-sided)	Exact Sig. (1-sided)
Pearson Chi-Square	3.829[a]	1	.050		
Continuity Correction[b]	2.548	1	.110		
Likelihood Ratio	3.989	1	.046		
Fisher's Exact Test				.073	.054
N of Valid Cases	63				

a. 2 cells (50.0%) have expected count less than 5. The minimum expected count is 4.29.

b. Computed only for a 2x2 table

Gottron's sign * PLantisynthetase

Crosstab

			PLantisynthetase		Total
			No	Yes	
Gottron's sign	No	Count	20	4	24
		% within PLantisynthetase	38.5%	36.4%	38.1%
		% of Total	31.7%	6.3%	38.1%
	Yes	Count	32	7	39
		% within PLantisynthetase	61.5%	63.6%	61.9%
		% of Total	50.8%	11.1%	61.9%
Total		Count	52	11	63
		% within PLantisynthetase	100.0%	100.0%	100.0%
		% of Total	82.5%	17.5%	100.0%

Chi-Square Tests

	Value	df	Asymp. Sig. (2-sided)	Exact Sig. (2-sided)	Exact Sig. (1-sided)
Pearson Chi-Square	.017[a]	1	.896		
Continuity Correction[b]	.000	1	1.000		
Likelihood Ratio	.017	1	.896		
Fisher's Exact Test				1.000	.590
N of Valid Cases	63				

a. 1 cells (25.0%) have expected count less than 5. The minimum expected count is 4.19.

b. Computed only for a 2x2 table

Gottron's sign * PLTIF1y

Crosstab

			PLTIF1y		Total
			No	Yes	
Gottron's sign	No	Count	20	4	24
		% within PLTIF1y	44.4%	22.2%	38.1%
		% of Total	31.7%	6.3%	38.1%
	Yes	Count	25	14	39
		% within PLTIF1y	55.6%	77.8%	61.9%
		% of Total	39.7%	22.2%	61.9%
Total		Count	45	18	63
		% within PLTIF1y	100.0%	100.0%	100.0%
		% of Total	71.4%	28.6%	100.0%

Chi-Square Tests

	Value	df	Asymp. Sig. (2-sided)	Exact Sig. (2-sided)	Exact Sig. (1-sided)
Pearson Chi-Square	2.692[a]	1	.101		
Continuity Correction[b]	1.832	1	.176		
Likelihood Ratio	2.835	1	.092		
Fisher's Exact Test				.152	.086
N of Valid Cases	63				

a. 0 cells (.0%) have expected count less than 5. The minimum expected count is 6.86.

b. Computed only for a 2x2 table

Gottron's sign * PLRo52

Crosstab

			PLRo52		Total
			No	Yes	
Gottron's sign	No	Count	18	6	24
		% within PLRo52	36.7%	42.9%	38.1%
		% of Total	28.6%	9.5%	38.1%
	Yes	Count	31	8	39
		% within PLRo52	63.3%	57.1%	61.9%
		% of Total	49.2%	12.7%	61.9%
Total		Count	49	14	63
		% within PLRo52	100.0%	100.0%	100.0%
		% of Total	77.8%	22.2%	100.0%

Chi-Square Tests

	Value	df	Asymp. Sig. (2-sided)	Exact Sig. (2-sided)	Exact Sig. (1-sided)
Pearson Chi-Square	.173[a]	1	.677		
Continuity Correction[b]	.011	1	.917		
Likelihood Ratio	.171	1	.679		
Fisher's Exact Test				.759	.453
N of Valid Cases	63				

a. 0 cells (.0%) have expected count less than 5. The minimum expected count is 5.33.

b. Computed only for a 2x2 table

Gottron's sign * PLMi2

Crosstab

			PLMi2		Total
			No	Yes	
Gottron's sign	No	Count	20	4	24
		% within PLMi2	37.0%	44.4%	38.1%
		% of Total	31.7%	6.3%	38.1%
	Yes	Count	34	5	39
		% within PLMi2	63.0%	55.6%	61.9%
		% of Total	54.0%	7.9%	61.9%
Total		Count	54	9	63
		% within PLMi2	100.0%	100.0%	100.0%
		% of Total	85.7%	14.3%	100.0%

Chi-Square Tests

	Value	df	Asymp. Sig. (2-sided)	Exact Sig. (2-sided)	Exact Sig. (1-sided)
Pearson Chi-Square	.179[a]	1	.672		
Continuity Correction[b]	.003	1	.958		
Likelihood Ratio	.177	1	.674		
Fisher's Exact Test				.721	.470
N of Valid Cases	63				

a. 1 cells (25.0%) have expected count less than 5. The minimum expected count is 3.43.

b. Computed only for a 2x2 table

Gottron's sign * PLMDA5

Crosstab

			PLMDA5		Total
			No	Yes	
Gottron's sign	No	Count	18	6	24
		% within PLMDA5	33.3%	66.7%	38.1%
		% of Total	28.6%	9.5%	38.1%
	Yes	Count	36	3	39
		% within PLMDA5	66.7%	33.3%	61.9%
		% of Total	57.1%	4.8%	61.9%
Total		Count	54	9	63
		% within PLMDA5	100.0%	100.0%	100.0%
		% of Total	85.7%	14.3%	100.0%

Chi-Square Tests

	Value	df	Asymp. Sig. (2-sided)	Exact Sig. (2-sided)	Exact Sig. (1-sided)
Pearson Chi-Square	3.635[a]	1	.057		
Continuity Correction[b]	2.359	1	.125		
Likelihood Ratio	3.530	1	.060		
Fisher's Exact Test				.073	.064
N of Valid Cases	63				

a. 1 cells (25.0%) have expected count less than 5. The minimum expected count is 3.43.

b. Computed only for a 2x2 table

Poikiloderma * PLantisynthetase

Crosstab

			PLantisynthetase		Total
			No	Yes	
Poikiloderma	No	Count	40	6	46
		% within PLantisynthetase	76.9%	54.5%	73.0%
		% of Total	63.5%	9.5%	73.0%
	Yes	Count	12	5	17
		% within PLantisynthetase	23.1%	45.5%	27.0%
		% of Total	19.0%	7.9%	27.0%
Total		Count	52	11	63
		% within PLantisynthetase	100.0%	100.0%	100.0%
		% of Total	82.5%	17.5%	100.0%

Chi-Square Tests

	Value	df	Asymp. Sig. (2-sided)	Exact Sig. (2-sided)	Exact Sig. (1-sided)
Pearson Chi-Square	2.308[a]	1	.129		
Continuity Correction[b]	1.312	1	.252		
Likelihood Ratio	2.131	1	.144		
Fisher's Exact Test				.149	.127
N of Valid Cases	63				

a. 1 cells (25.0%) have expected count less than 5. The minimum expected count is 2.97.

b. Computed only for a 2x2 table

Poikiloderma * PLTIF1y

Crosstab

			PLTIF1y		Total
			No	Yes	
Poikiloderma	No	Count	37	9	46
		% within PLTIF1y	82.2%	50.0%	73.0%
		% of Total	58.7%	14.3%	73.0%
	Yes	Count	8	9	17
		% within PLTIF1y	17.8%	50.0%	27.0%
		% of Total	12.7%	14.3%	27.0%
Total		Count	45	18	63
		% within PLTIF1y	100.0%	100.0%	100.0%
		% of Total	71.4%	28.6%	100.0%

Chi-Square Tests

	Value	df	Asymp. Sig. (2-sided)	Exact Sig. (2-sided)	Exact Sig. (1-sided)
Pearson Chi-Square	6.775[a]	1	.009		
Continuity Correction[b]	5.239	1	.022		
Likelihood Ratio	6.397	1	.011		
Fisher's Exact Test				.014	.013
N of Valid Cases	63				

a. 1 cells (25.0%) have expected count less than 5. The minimum expected count is 4.86.

b. Computed only for a 2x2 table

Poikiloderma * PLRo52

Crosstab

			PLRo52		Total
			No	Yes	
Poikiloderma	No	Count	38	8	46
		% within PLRo52	77.6%	57.1%	73.0%
		% of Total	60.3%	12.7%	73.0%
	Yes	Count	11	6	17
		% within PLRo52	22.4%	42.9%	27.0%
		% of Total	17.5%	9.5%	27.0%
Total		Count	49	14	63
		% within PLRo52	100.0%	100.0%	100.0%
		% of Total	77.8%	22.2%	100.0%

Chi-Square Tests

	Value	df	Asymp. Sig. (2-sided)	Exact Sig. (2-sided)	Exact Sig. (1-sided)
Pearson Chi-Square	2.302[a]	1	.129		
Continuity Correction[b]	1.383	1	.240		
Likelihood Ratio	2.161	1	.142		
Fisher's Exact Test				.174	.121
N of Valid Cases	63				

a. 1 cells (25.0%) have expected count less than 5. The minimum expected count is 3.78.

b. Computed only for a 2x2 table

Poikiloderma * PLMi2

Crosstab

			PLMi2		Total
			No	Yes	
Poikiloderma	No	Count	39	7	46
		% within PLMi2	72.2%	77.8%	73.0%
		% of Total	61.9%	11.1%	73.0%
	Yes	Count	15	2	17
		% within PLMi2	27.8%	22.2%	27.0%
		% of Total	23.8%	3.2%	27.0%
Total		Count	54	9	63
		% within PLMi2	100.0%	100.0%	100.0%
		% of Total	85.7%	14.3%	100.0%

Chi-Square Tests

	Value	df	Asymp. Sig. (2-sided)	Exact Sig. (2-sided)	Exact Sig. (1-sided)
Pearson Chi-Square	.121[a]	1	.728		
Continuity Correction[b]	.000	1	1.000		
Likelihood Ratio	.125	1	.724		
Fisher's Exact Test				1.000	.542
N of Valid Cases	63				

a. 1 cells (25.0%) have expected count less than 5. The minimum expected count is 2.43.

b. Computed only for a 2x2 table

Poikiloderma * PLMDA5

Crosstab

			PLMDA5		Total
			No	Yes	
Poikiloderma	No	Count	37	9	46
		% within PLMDA5	68.5%	100.0%	73.0%
		% of Total	58.7%	14.3%	73.0%
	Yes	Count	17	0	17
		% within PLMDA5	31.5%	0.0%	27.0%
		% of Total	27.0%	0.0%	27.0%
Total		Count	54	9	63
		% within PLMDA5	100.0%	100.0%	100.0%
		% of Total	85.7%	14.3%	100.0%

Chi-Square Tests

	Value	df	Asymp. Sig. (2-sided)	Exact Sig. (2-sided)	Exact Sig. (1-sided)
Pearson Chi-Square	3.880[a]	1	.049		
Continuity Correction[b]	2.447	1	.118		
Likelihood Ratio	6.198	1	.013		
Fisher's Exact Test				.098	.047
N of Valid Cases	63				

a. 1 cells (25.0%) have expected count less than 5. The minimum expected count is 2.43.

b. Computed only for a 2x2 table

Subcutaneous calcification * PLantisynthetase

Crosstab

			PLantisynthetase		Total
			No	Yes	
Subcutaneous calcification	No	Count	51	9	60
		% within PLantisynthetase	98.1%	81.8%	95.2%
		% of Total	81.0%	14.3%	95.2%
	Yes	Count	1	2	3
		% within PLantisynthetase	1.9%	18.2%	4.8%
		% of Total	1.6%	3.2%	4.8%
Total		Count	52	11	63
		% within PLantisynthetase	100.0%	100.0%	100.0%
		% of Total	82.5%	17.5%	100.0%

Chi-Square Tests

	Value	df	Asymp. Sig. (2-sided)	Exact Sig. (2-sided)	Exact Sig. (1-sided)
Pearson Chi-Square	5.292[a]	1	.021		
Continuity Correction[b]	2.314	1	.128		
Likelihood Ratio	3.808	1	.051		
Fisher's Exact Test				.076	.076
N of Valid Cases	63				

a. 2 cells (50.0%) have expected count less than 5. The minimum expected count is .52.

b. Computed only for a 2x2 table

Subcutaneous calcification * PLTIF1y

Crosstab

			PLTIF1y		
			No	Yes	Total
Subcutaneous calcification	No	Count	43	17	60
		% within PLTIF1y	95.6%	94.4%	95.2%
		% of Total	68.3%	27.0%	95.2%
	Yes	Count	2	1	3
		% within PLTIF1y	4.4%	5.6%	4.8%
		% of Total	3.2%	1.6%	4.8%
Total		Count	45	18	63
		% within PLTIF1y	100.0%	100.0%	100.0%
		% of Total	71.4%	28.6%	100.0%

Chi-Square Tests

	Value	df	Asymp. Sig. (2-sided)	Exact Sig. (2-sided)	Exact Sig. (1-sided)
Pearson Chi-Square	.035[a]	1	.852		
Continuity Correction[b]	.000	1	1.000		
Likelihood Ratio	.034	1	.854		
Fisher's Exact Test				1.000	.643
N of Valid Cases	63				

a. 2 cells (50.0%) have expected count less than 5. The minimum expected count is .86.

b. Computed only for a 2x2 table

Subcutaneous calcification * PLRo52

Crosstab

			PLRo52		Total
			No	Yes	
Subcutaneous calcification	No	Count	46	14	60
		% within PLRo52	93.9%	100.0%	95.2%
		% of Total	73.0%	22.2%	95.2%
	Yes	Count	3	0	3
		% within PLRo52	6.1%	0.0%	4.8%
		% of Total	4.8%	0.0%	4.8%
Total		Count	49	14	63
		% within PLRo52	100.0%	100.0%	100.0%
		% of Total	77.8%	22.2%	100.0%

Chi-Square Tests

	Value	df	Asymp. Sig. (2-sided)	Exact Sig. (2-sided)	Exact Sig. (1-sided)
Pearson Chi-Square	.900[a]	1	.343		
Continuity Correction[b]	.056	1	.813		
Likelihood Ratio	1.550	1	.213		
Fisher's Exact Test				1.000	.464
N of Valid Cases	63				

a. 2 cells (50.0%) have expected count less than 5. The minimum expected count is .67.

b. Computed only for a 2x2 table

Subcutaneous calcification * PLMi2

Crosstab

			PLMi2		Total
			No	Yes	
Subcutaneous calcification	No	Count	51	9	60
		% within PLMi2	94.4%	100.0%	95.2%
		% of Total	81.0%	14.3%	95.2%
	Yes	Count	3	0	3
		% within PLMi2	5.6%	0.0%	4.8%
		% of Total	4.8%	0.0%	4.8%
Total		Count	54	9	63
		% within PLMi2	100.0%	100.0%	100.0%
		% of Total	85.7%	14.3%	100.0%

Chi-Square Tests

	Value	df	Asymp. Sig. (2-sided)	Exact Sig. (2-sided)	Exact Sig. (1-sided)
Pearson Chi-Square	.525[a]	1	.469		
Continuity Correction[b]	.000	1	1.000		
Likelihood Ratio	.950	1	.330		
Fisher's Exact Test				1.000	.625
N of Valid Cases	63				

a. 2 cells (50.0%) have expected count less than 5. The minimum expected count is .43.

b. Computed only for a 2x2 table

Subcutaneous calcification * PLMDA5

Crosstab

			PLMDA5		Total
			No	Yes	
Subcutaneous calcification	No	Count	52	8	60
		% within PLMDA5	96.3%	88.9%	95.2%
		% of Total	82.5%	12.7%	95.2%
	Yes	Count	2	1	3
		% within PLMDA5	3.7%	11.1%	4.8%
		% of Total	3.2%	1.6%	4.8%
Total		Count	54	9	63
		% within PLMDA5	100.0%	100.0%	100.0%
		% of Total	85.7%	14.3%	100.0%

Chi-Square Tests

	Value	df	Asymp. Sig. (2-sided)	Exact Sig. (2-sided)	Exact Sig. (1-sided)
Pearson Chi-Square	.933[a]	1	.334		
Continuity Correction[b]	.015	1	.904		
Likelihood Ratio	.735	1	.391		
Fisher's Exact Test				.375	.375
N of Valid Cases	63				

a. 2 cells (50.0%) have expected count less than 5. The minimum expected count is .43.

b. Computed only for a 2x2 table

Mechanic's hands * PLantisynthetase

Crosstab

			PLantisynthetase		Total
			No	Yes	
Mechanic’s hands	No	Count	48	8	56
		% within PLantisynthetase	92.3%	72.7%	88.9%
		% of Total	76.2%	12.7%	88.9%
	Yes	Count	4	3	7
		% within PLantisynthetase	7.7%	27.3%	11.1%
		% of Total	6.3%	4.8%	11.1%
Total		Count	52	11	63
		% within PLantisynthetase	100.0%	100.0%	100.0%
		% of Total	82.5%	17.5%	100.0%

Chi-Square Tests

	Value	df	Asymp. Sig. (2-sided)	Exact Sig. (2-sided)	Exact Sig. (1-sided)
Pearson Chi-Square	3.524[a]	1	.060		
Continuity Correction[b]	1.821	1	.177		
Likelihood Ratio	2.858	1	.091		
Fisher's Exact Test				.095	.095
N of Valid Cases	63				

a. 1 cells (25.0%) have expected count less than 5. The minimum expected count is 1.22.

b. Computed only for a 2x2 table

Mechanic’s hands * PLTIF1y

Crosstab

			PLTIF1y		Total
			No	Yes	
Mechanic’s hands	No	Count	41	15	56
		% within PLTIF1y	91.1%	83.3%	88.9%
		% of Total	65.1%	23.8%	88.9%
	Yes	Count	4	3	7
		% within PLTIF1y	8.9%	16.7%	11.1%
		% of Total	6.3%	4.8%	11.1%
Total		Count	45	18	63
		% within PLTIF1y	100.0%	100.0%	100.0%
		% of Total	71.4%	28.6%	100.0%

Chi-Square Tests

	Value	df	Asymp. Sig. (2-sided)	Exact Sig. (2-sided)	Exact Sig. (1-sided)
Pearson Chi-Square	.788[a]	1	.375		
Continuity Correction[b]	.197	1	.657		
Likelihood Ratio	.736	1	.391		
Fisher's Exact Test				.397	.315
N of Valid Cases	63				

a. 1 cells (25.0%) have expected count less than 5. The minimum expected count is 2.00.

b. Computed only for a 2x2 table

Mechanic's hands * PLRo52

Crosstab

			PLRo52		Total
			No	Yes	
Mechanic's hands	No	Count	45	11	56
		% within PLRo52	91.8%	78.6%	88.9%
		% of Total	71.4%	17.5%	88.9%
	Yes	Count	4	3	7
		% within PLRo52	8.2%	21.4%	11.1%
		% of Total	6.3%	4.8%	11.1%
Total		Count	49	14	63
		% within PLRo52	100.0%	100.0%	100.0%
		% of Total	77.8%	22.2%	100.0%

Chi-Square Tests

	Value	df	Asymp. Sig. (2-sided)	Exact Sig. (2-sided)	Exact Sig. (1-sided)
Pearson Chi-Square	1.940[a]	1	.164		
Continuity Correction[b]	.829	1	.362		
Likelihood Ratio	1.696	1	.193		
Fisher's Exact Test				.177	.177
N of Valid Cases	63				

a. 1 cells (25.0%) have expected count less than 5. The minimum expected count is 1.56.

b. Computed only for a 2x2 table

Mechanic's hands * PLMi2

Crosstab

			PLMi2		Total
			No	Yes	
Mechanic’s hands	No	Count	47	9	56
		% within PLMi2	87.0%	100.0%	88.9%
		% of Total	74.6%	14.3%	88.9%
	Yes	Count	7	0	7
		% within PLMi2	13.0%	0.0%	11.1%
		% of Total	11.1%	0.0%	11.1%
Total		Count	54	9	63
		% within PLMi2	100.0%	100.0%	100.0%
		% of Total	85.7%	14.3%	100.0%

Chi-Square Tests

	Value	df	Asymp. Sig. (2-sided)	Exact Sig. (2-sided)	Exact Sig. (1-sided)
Pearson Chi-Square	1.313[a]	1	.252		
Continuity Correction[b]	.328	1	.567		
Likelihood Ratio	2.299	1	.129		
Fisher's Exact Test				.580	.320
N of Valid Cases	63				

a. 1 cells (25.0%) have expected count less than 5. The minimum expected count is 1.00.

b. Computed only for a 2x2 table

Mechanic’s hands * PLMDA5

Crosstab

			PLMDA5		Total
			No	Yes	
Mechanic’s hands	No	Count	48	8	56
		% within PLMDA5	88.9%	88.9%	88.9%
		% of Total	76.2%	12.7%	88.9%
	Yes	Count	6	1	7
		% within PLMDA5	11.1%	11.1%	11.1%
		% of Total	9.5%	1.6%	11.1%
Total		Count	54	9	63
		% within PLMDA5	100.0%	100.0%	100.0%
		% of Total	85.7%	14.3%	100.0%

Chi-Square Tests

	Value	df	Asymp. Sig. (2-sided)	Exact Sig. (2-sided)	Exact Sig. (1-sided)
Pearson Chi-Square	.000[a]	1	1.000		
Continuity Correction[b]	.000	1	1.000		
Likelihood Ratio	.000	1	1.000		
Fisher's Exact Test				1.000	.740
N of Valid Cases	63				

a. 1 cells (25.0%) have expected count less than 5. The minimum expected count is 1.00.

b. Computed only for a 2x2 table

Periungual fissures * PLantisynthetase

Crosstab

			PLantisynthetase		
			No	Yes	Total
Periungual fissures	No	Count	39	9	48
		% within PLantisynthetase	75.0%	81.8%	76.2%
		% of Total	61.9%	14.3%	76.2%
	Yes	Count	13	2	15
		% within PLantisynthetase	25.0%	18.2%	23.8%
		% of Total	20.6%	3.2%	23.8%
Total		Count	52	11	63
		% within PLantisynthetase	100.0%	100.0%	100.0%
		% of Total	82.5%	17.5%	100.0%

Chi-Square Tests

	Value	df	Asymp. Sig. (2-sided)	Exact Sig. (2-sided)	Exact Sig. (1-sided)
Pearson Chi-Square	.233[a]	1	.630		
Continuity Correction[b]	.009	1	.926		
Likelihood Ratio	.244	1	.621		
Fisher's Exact Test				1.000	.482
N of Valid Cases	63				

a. 1 cells (25.0%) have expected count less than 5. The minimum expected count is 2.62.

b. Computed only for a 2x2 table

Periungual fissures * PLTIF1y

Crosstab

			PLTIF1y		Total
			No	Yes	
Periungual fissures	No	Count	37	11	48
		% within PLTIF1y	82.2%	61.1%	76.2%
		% of Total	58.7%	17.5%	76.2%
	Yes	Count	8	7	15
		% within PLTIF1y	17.8%	38.9%	23.8%
		% of Total	12.7%	11.1%	23.8%
Total		Count	45	18	63
		% within PLTIF1y	100.0%	100.0%	100.0%
		% of Total	71.4%	28.6%	100.0%

Chi-Square Tests

	Value	df	Asymp. Sig. (2-sided)	Exact Sig. (2-sided)	Exact Sig. (1-sided)
Pearson Chi-Square	3.159[a]	1	.076		
Continuity Correction[b]	2.102	1	.147		
Likelihood Ratio	2.981	1	.084		
Fisher's Exact Test				.104	.076
N of Valid Cases	63				

a. 1 cells (25.0%) have expected count less than 5. The minimum expected count is 4.29.

b. Computed only for a 2x2 table

Periungual fissures * PLRo52

Crosstab

			PLRo52		Total
			No	Yes	
Periungual fissures	No	Count	39	9	48
		% within PLRo52	79.6%	64.3%	76.2%
		% of Total	61.9%	14.3%	76.2%
	Yes	Count	10	5	15
		% within PLRo52	20.4%	35.7%	23.8%
		% of Total	15.9%	7.9%	23.8%
Total		Count	49	14	63
		% within PLRo52	100.0%	100.0%	100.0%
		% of Total	77.8%	22.2%	100.0%

Chi-Square Tests

	Value	df	Asymp. Sig. (2-sided)	Exact Sig. (2-sided)	Exact Sig. (1-sided)
Pearson Chi-Square	1.406[a]	1	.236		
Continuity Correction[b]	.689	1	.406		
Likelihood Ratio	1.320	1	.251		
Fisher's Exact Test				.291	.200
N of Valid Cases	63				

a. 1 cells (25.0%) have expected count less than 5. The minimum expected count is 3.33.

b. Computed only for a 2x2 table

Periungual fissures * PLMi2

Crosstab

			PLMi2		Total
			No	Yes	
Periungual fissures	No	Count	40	8	48
		% within PLMi2	74.1%	88.9%	76.2%
		% of Total	63.5%	12.7%	76.2%
	Yes	Count	14	1	15
		% within PLMi2	25.9%	11.1%	23.8%
		% of Total	22.2%	1.6%	23.8%
Total		Count	54	9	63
		% within PLMi2	100.0%	100.0%	100.0%
		% of Total	85.7%	14.3%	100.0%

Chi-Square Tests

	Value	df	Asymp. Sig. (2-sided)	Exact Sig. (2-sided)	Exact Sig. (1-sided)
Pearson Chi-Square	.933[a]	1	.334		
Continuity Correction[b]	.295	1	.587		
Likelihood Ratio	1.073	1	.300		
Fisher's Exact Test				.673	.310
N of Valid Cases	63				

a. 1 cells (25.0%) have expected count less than 5. The minimum expected count is 2.14.

b. Computed only for a 2x2 table

Periungual fissures * PLMDA5

Crosstab

			PLMDA5		Total
			No	Yes	
Periungual fissures	No	Count	40	8	48
		% within PLMDA5	74.1%	88.9%	76.2%
		% of Total	63.5%	12.7%	76.2%
	Yes	Count	14	1	15
		% within PLMDA5	25.9%	11.1%	23.8%
		% of Total	22.2%	1.6%	23.8%
Total		Count	54	9	63
		% within PLMDA5	100.0%	100.0%	100.0%
		% of Total	85.7%	14.3%	100.0%

Chi-Square Tests

	Value	df	Asymp. Sig. (2-sided)	Exact Sig. (2-sided)	Exact Sig. (1-sided)
Pearson Chi-Square	.933[a]	1	.334		
Continuity Correction[b]	.295	1	.587		
Likelihood Ratio	1.073	1	.300		
Fisher's Exact Test				.673	.310
N of Valid Cases	63				

a. 1 cells (25.0%) have expected count less than 5. The minimum expected count is 2.14.

b. Computed only for a 2x2 table

Periungual telangiectasia * PLantisynthetase

Crosstab

			PLantisynthetase		Total
			No	Yes	
Periungual telangiectasia	No	Count	31	6	37
		% within PLantisynthetase	59.6%	54.5%	58.7%
		% of Total	49.2%	9.5%	58.7%
	Yes	Count	21	5	26
		% within PLantisynthetase	40.4%	45.5%	41.3%
		% of Total	33.3%	7.9%	41.3%
Total		Count	52	11	63
		% within PLantisynthetase	100.0%	100.0%	100.0%
		% of Total	82.5%	17.5%	100.0%

Chi-Square Tests

	Value	df	Asymp. Sig. (2-sided)	Exact Sig. (2-sided)	Exact Sig. (1-sided)
Pearson Chi-Square	.096[a]	1	.756		
Continuity Correction[b]	.000	1	1.000		
Likelihood Ratio	.096	1	.757		
Fisher's Exact Test				.750	.505
N of Valid Cases	63				

a. 1 cells (25.0%) have expected count less than 5. The minimum expected count is 4.54.

b. Computed only for a 2x2 table

Periungual telangiectasia * PLTIF1y

Crosstab

			PLTIF1y		
			No	Yes	Total
Periungual telangiectasia	No	Count	28	9	37
		% within PLTIF1y	62.2%	50.0%	58.7%
		% of Total	44.4%	14.3%	58.7%
	Yes	Count	17	9	26
		% within PLTIF1y	37.8%	50.0%	41.3%
		% of Total	27.0%	14.3%	41.3%
Total		Count	45	18	63
		% within PLTIF1y	100.0%	100.0%	100.0%
		% of Total	71.4%	28.6%	100.0%

Chi-Square Tests

	Value	df	Asymp. Sig. (2-sided)	Exact Sig. (2-sided)	Exact Sig. (1-sided)
Pearson Chi-Square	.792[a]	1	.373		
Continuity Correction[b]	.368	1	.544		
Likelihood Ratio	.786	1	.375		
Fisher's Exact Test				.408	.271
N of Valid Cases	63				

a. 0 cells (.0%) have expected count less than 5. The minimum expected count is 7.43.

b. Computed only for a 2x2 table

Periungual telangiectasia * PLRo52

Crosstab

			PLRo52		Total
			No	Yes	
Periungual telangiectasia	No	Count	29	8	37
		% within PLRo52	59.2%	57.1%	58.7%
		% of Total	46.0%	12.7%	58.7%
	Yes	Count	20	6	26
		% within PLRo52	40.8%	42.9%	41.3%
		% of Total	31.7%	9.5%	41.3%
Total		Count	49	14	63
		% within PLRo52	100.0%	100.0%	100.0%
		% of Total	77.8%	22.2%	100.0%

Chi-Square Tests

	Value	df	Asymp. Sig. (2-sided)	Exact Sig. (2-sided)	Exact Sig. (1-sided)
Pearson Chi-Square	.019[a]	1	.891		
Continuity Correction[b]	.000	1	1.000		
Likelihood Ratio	.019	1	.891		
Fisher's Exact Test				1.000	.564
N of Valid Cases	63				

a. 0 cells (.0%) have expected count less than 5. The minimum expected count is 5.78.

b. Computed only for a 2x2 table

Periungual telangiectasia * PLMi2

Crosstab

			PLMi2		Total
			No	Yes	
Periungual telangiectasia	No	Count	32	5	37
		% within PLMi2	59.3%	55.6%	58.7%
		% of Total	50.8%	7.9%	58.7%
	Yes	Count	22	4	26
		% within PLMi2	40.7%	44.4%	41.3%
		% of Total	34.9%	6.3%	41.3%
Total		Count	54	9	63
		% within PLMi2	100.0%	100.0%	100.0%
		% of Total	85.7%	14.3%	100.0%

Chi-Square Tests

	Value	df	Asymp. Sig. (2-sided)	Exact Sig. (2-sided)	Exact Sig. (1-sided)
Pearson Chi-Square	.044[a]	1	.834		
Continuity Correction[b]	.000	1	1.000		
Likelihood Ratio	.043	1	.835		
Fisher's Exact Test				1.000	.556
N of Valid Cases	63				

a. 1 cells (25.0%) have expected count less than 5. The minimum expected count is 3.71.

b. Computed only for a 2x2 table

Periungual telangiectasia * PLMDA5

Crosstab

			PLMDA5		Total
			No	Yes	
Periungual telangiectasia	No	Count	31	6	37
		% within PLMDA5	57.4%	66.7%	58.7%
		% of Total	49.2%	9.5%	58.7%
	Yes	Count	23	3	26
		% within PLMDA5	42.6%	33.3%	41.3%
		% of Total	36.5%	4.8%	41.3%
Total		Count	54	9	63
		% within PLMDA5	100.0%	100.0%	100.0%
		% of Total	85.7%	14.3%	100.0%

Chi-Square Tests

	Value	df	Asymp. Sig. (2-sided)	Exact Sig. (2-sided)	Exact Sig. (1-sided)
Pearson Chi-Square	.273[a]	1	.601		
Continuity Correction[b]	.025	1	.875		
Likelihood Ratio	.278	1	.598		
Fisher's Exact Test				.725	.444
N of Valid Cases	63				

a. 1 cells (25.0%) have expected count less than 5. The minimum expected count is 3.71.

b. Computed only for a 2x2 table

Periungual haemorrhage * PLantisynthetase

Crosstab

			PLantisynthetase		Total
			No	Yes	
Periungual haemorrhage	No	Count	46	11	57
		% within PLantisynthetase	88.5%	100.0%	90.5%
		% of Total	73.0%	17.5%	90.5%
	Yes	Count	6	0	6
		% within PLantisynthetase	11.5%	0.0%	9.5%
		% of Total	9.5%	0.0%	9.5%
Total		Count	52	11	63
		% within PLantisynthetase	100.0%	100.0%	100.0%
		% of Total	82.5%	17.5%	100.0%

Chi-Square Tests

	Value	df	Asymp. Sig. (2-sided)	Exact Sig. (2-sided)	Exact Sig. (1-sided)
Pearson Chi-Square	1.403[a]	1	.236		
Continuity Correction[b]	.383	1	.536		
Likelihood Ratio	2.433	1	.119		
Fisher's Exact Test				.579	.300
N of Valid Cases	63				

a. 2 cells (50.0%) have expected count less than 5. The minimum expected count is 1.05.

b. Computed only for a 2x2 table

Periungual haemorrhage * PLTIF1y

Crosstab

			PLTIF1y		Total
			No	Yes	
Periungual haemorrhage	No	Count	43	14	57
		% within PLTIF1y	95.6%	77.8%	90.5%
		% of Total	68.3%	22.2%	90.5%
	Yes	Count	2	4	6
		% within PLTIF1y	4.4%	22.2%	9.5%
		% of Total	3.2%	6.3%	9.5%
Total		Count	45	18	63
		% within PLTIF1y	100.0%	100.0%	100.0%
		% of Total	71.4%	28.6%	100.0%

Chi-Square Tests

	Value	df	Asymp. Sig. (2-sided)	Exact Sig. (2-sided)	Exact Sig. (1-sided)
Pearson Chi-Square	4.716[a]	1	.030		
Continuity Correction[b]	2.878	1	.090		
Likelihood Ratio	4.193	1	.041		
Fisher's Exact Test				.051	.051
N of Valid Cases	63				

a. 2 cells (50.0%) have expected count less than 5. The minimum expected count is 1.71.

b. Computed only for a 2x2 table

Periungual haemorrhage * PLRo52

Crosstab

			PLRo52		
			No	Yes	Total
Periungual haemorrhage	No	Count	45	12	57
		% within PLRo52	91.8%	85.7%	90.5%
		% of Total	71.4%	19.0%	90.5%
	Yes	Count	4	2	6
		% within PLRo52	8.2%	14.3%	9.5%
		% of Total	6.3%	3.2%	9.5%
Total		Count	49	14	63
		% within PLRo52	100.0%	100.0%	100.0%
		% of Total	77.8%	22.2%	100.0%

Chi-Square Tests

	Value	df	Asymp. Sig. (2-sided)	Exact Sig. (2-sided)	Exact Sig. (1-sided)
Pearson Chi-Square	.474[a]	1	.491		
Continuity Correction[b]	.030	1	.863		
Likelihood Ratio	.434	1	.510		
Fisher's Exact Test				.607	.401
N of Valid Cases	63				

a. 2 cells (50.0%) have expected count less than 5. The minimum expected count is 1.33.

b. Computed only for a 2x2 table

Periungual haemorrhage * PLMi2

Crosstab

			PLMi2		Total
			No	Yes	
Periungual haemorrhage	No	Count	49	8	57
		% within PLMi2	90.7%	88.9%	90.5%
		% of Total	77.8%	12.7%	90.5%
	Yes	Count	5	1	6
		% within PLMi2	9.3%	11.1%	9.5%
		% of Total	7.9%	1.6%	9.5%
Total		Count	54	9	63
		% within PLMi2	100.0%	100.0%	100.0%
		% of Total	85.7%	14.3%	100.0%

Chi-Square Tests

	Value	df	Asymp. Sig. (2-sided)	Exact Sig. (2-sided)	Exact Sig. (1-sided)
Pearson Chi-Square	.031[a]	1	.861		
Continuity Correction[b]	.000	1	1.000		
Likelihood Ratio	.030	1	.864		
Fisher's Exact Test				1.000	.620
N of Valid Cases	63				

a. 1 cells (25.0%) have expected count less than 5. The minimum expected count is .86.

b. Computed only for a 2x2 table

Periungual haemorrhage * PLMDA5

Crosstab

			PLMDA5		Total
			No	Yes	
Periungual haemorrhage	No	Count	49	8	57
		% within PLMDA5	90.7%	88.9%	90.5%
		% of Total	77.8%	12.7%	90.5%
	Yes	Count	5	1	6
		% within PLMDA5	9.3%	11.1%	9.5%
		% of Total	7.9%	1.6%	9.5%
Total		Count	54	9	63
		% within PLMDA5	100.0%	100.0%	100.0%
		% of Total	85.7%	14.3%	100.0%

Chi-Square Tests

	Value	df	Asymp. Sig. (2-sided)	Exact Sig. (2-sided)	Exact Sig. (1-sided)
Pearson Chi-Square	.031[a]	1	.861		
Continuity Correction[b]	.000	1	1.000		
Likelihood Ratio	.030	1	.864		
Fisher's Exact Test				1.000	.620
N of Valid Cases	63				

a. 1 cells (25.0%) have expected count less than 5. The minimum expected count is .86.

b. Computed only for a 2x2 table

Raynaud's phenomenon * PLantisynthetase

Crosstab

			PLantisynthetase		Total
			No	Yes	
Raynaud's phenomenon	No	Count	49	5	54
		% within PLantisynthetase	94.2%	45.5%	85.7%
		% of Total	77.8%	7.9%	85.7%
	Yes	Count	3	6	9
		% within PLantisynthetase	5.8%	54.5%	14.3%
		% of Total	4.8%	9.5%	14.3%
Total		Count	52	11	63
		% within PLantisynthetase	100.0%	100.0%	100.0%
		% of Total	82.5%	17.5%	100.0%

Chi-Square Tests

	Value	df	Asymp. Sig. (2-sided)	Exact Sig. (2-sided)	Exact Sig. (1-sided)
Pearson Chi-Square	17.641[a]	1	.000		
Continuity Correction[b]	13.882	1	.000		
Likelihood Ratio	13.577	1	.000		
Fisher's Exact Test				.000	.000
N of Valid Cases	63				

a. 1 cells (25.0%) have expected count less than 5. The minimum expected count is 1.57.

b. Computed only for a 2x2 table

Raynaud's phenomenon * PLTIF1y

Crosstab

			PLTIF1y		Total
			No	Yes	
Raynaud's phenomenon	No	Count	39	15	54
		% within PLTIF1y	86.7%	83.3%	85.7%
		% of Total	61.9%	23.8%	85.7%
	Yes	Count	6	3	9
		% within PLTIF1y	13.3%	16.7%	14.3%
		% of Total	9.5%	4.8%	14.3%
Total		Count	45	18	63
		% within PLTIF1y	100.0%	100.0%	100.0%
		% of Total	71.4%	28.6%	100.0%

Chi-Square Tests

	Value	df	Asymp. Sig. (2-sided)	Exact Sig. (2-sided)	Exact Sig. (1-sided)
Pearson Chi-Square	.117[a]	1	.733		
Continuity Correction[b]	.000	1	1.000		
Likelihood Ratio	.114	1	.736		
Fisher's Exact Test				.707	.505
N of Valid Cases	63				

a. 1 cells (25.0%) have expected count less than 5. The minimum expected count is 2.57.

b. Computed only for a 2x2 table

Raynaud's phenomenon * PLRo52

Crosstab

			PLRo52		Total
			No	Yes	
Raynaud's phenomenon	No	Count	41	13	54
		% within PLRo52	83.7%	92.9%	85.7%
		% of Total	65.1%	20.6%	85.7%
	Yes	Count	8	1	9
		% within PLRo52	16.3%	7.1%	14.3%
		% of Total	12.7%	1.6%	14.3%
Total		Count	49	14	63
		% within PLRo52	100.0%	100.0%	100.0%
		% of Total	77.8%	22.2%	100.0%

Chi-Square Tests

	Value	df	Asymp. Sig. (2-sided)	Exact Sig. (2-sided)	Exact Sig. (1-sided)
Pearson Chi-Square	.750[a]	1	.386		
Continuity Correction[b]	.188	1	.665		
Likelihood Ratio	.855	1	.355		
Fisher's Exact Test				.670	.354
N of Valid Cases	63				

a. 1 cells (25.0%) have expected count less than 5. The minimum expected count is 2.00.

b. Computed only for a 2x2 table

Raynaud’s phenomenon * PLMi2

Crosstab

			PLMi2		Total
			No	Yes	
Raynaud’s phenomenon	No	Count	47	7	54
		% within PLMi2	87.0%	77.8%	85.7%
		% of Total	74.6%	11.1%	85.7%
	Yes	Count	7	2	9
		% within PLMi2	13.0%	22.2%	14.3%
		% of Total	11.1%	3.2%	14.3%
Total		Count	54	9	63
		% within PLMi2	100.0%	100.0%	100.0%
		% of Total	85.7%	14.3%	100.0%

Chi-Square Tests

	Value	df	Asymp. Sig. (2-sided)	Exact Sig. (2-sided)	Exact Sig. (1-sided)
Pearson Chi-Square	.540[a]	1	.462		
Continuity Correction[b]	.049	1	.825		
Likelihood Ratio	.486	1	.486		
Fisher's Exact Test				.604	.380
N of Valid Cases	63				

a. 1 cells (25.0%) have expected count less than 5. The minimum expected count is 1.29.

b. Computed only for a 2x2 table

Raynaud’s phenomenon * PLMDA5

Crosstab

			PLMDA5		Total
			No	Yes	
Raynaud's phenomenon	No	Count	46	8	54
		% within PLMDA5	85.2%	88.9%	85.7%
		% of Total	73.0%	12.7%	85.7%
	Yes	Count	8	1	9
		% within PLMDA5	14.8%	11.1%	14.3%
		% of Total	12.7%	1.6%	14.3%
Total		Count	54	9	63
		% within PLMDA5	100.0%	100.0%	100.0%
		% of Total	85.7%	14.3%	100.0%

Chi-Square Tests

	Value	df	Asymp. Sig. (2-sided)	Exact Sig. (2-sided)	Exact Sig. (1-sided)
Pearson Chi-Square	.086[a]	1	.769		
Continuity Correction[b]	.000	1	1.000		
Likelihood Ratio	.091	1	.762		
Fisher's Exact Test				1.000	.620
N of Valid Cases	63				

a. 1 cells (25.0%) have expected count less than 5. The minimum expected count is 1.29.

b. Computed only for a 2x2 table

Skin ulcers * PLantisynthetase

Crosstab

			PLantisynthetase		Total
			No	Yes	
Skin ulcers	No	Count	48	9	57
		% within PLantisynthetase	92.3%	81.8%	90.5%
		% of Total	76.2%	14.3%	90.5%
	Yes	Count	4	2	6
		% within PLantisynthetase	7.7%	18.2%	9.5%
		% of Total	6.3%	3.2%	9.5%
Total		Count	52	11	63
		% within PLantisynthetase	100.0%	100.0%	100.0%
		% of Total	82.5%	17.5%	100.0%

Chi-Square Tests

	Value	df	Asymp. Sig. (2-sided)	Exact Sig. (2-sided)	Exact Sig. (1-sided)
Pearson Chi-Square	1.159[a]	1	.282		
Continuity Correction[b]	.262	1	.609		
Likelihood Ratio	.991	1	.319		
Fisher's Exact Test				.280	.280
N of Valid Cases	63				

a. 2 cells (50.0%) have expected count less than 5. The minimum expected count is 1.05.

b. Computed only for a 2x2 table

Skin ulcers * PLTIF1y

Crosstab

			PLTIF1y		
			No	Yes	Total
Skin ulcers	No	Count	42	15	57
		% within PLTIF1y	93.3%	83.3%	90.5%
		% of Total	66.7%	23.8%	90.5%
	Yes	Count	3	3	6
		% within PLTIF1y	6.7%	16.7%	9.5%
		% of Total	4.8%	4.8%	9.5%
Total		Count	45	18	63
		% within PLTIF1y	100.0%	100.0%	100.0%
		% of Total	71.4%	28.6%	100.0%

Chi-Square Tests

	Value	df	Asymp. Sig. (2-sided)	Exact Sig. (2-sided)	Exact Sig. (1-sided)
Pearson Chi-Square	1.492[a]	1	.222		
Continuity Correction[b]	.557	1	.455		
Likelihood Ratio	1.362	1	.243		
Fisher's Exact Test				.341	.221
N of Valid Cases	63				

a. 2 cells (50.0%) have expected count less than 5. The minimum expected count is 1.71.

b. Computed only for a 2x2 table

Skin ulcers * PLRo52

Crosstab

			PLRo52		Total
			No	Yes	
Skin ulcers	No	Count	43	14	57
		% within PLRo52	87.8%	100.0%	90.5%
		% of Total	68.3%	22.2%	90.5%
	Yes	Count	6	0	6
		% within PLRo52	12.2%	0.0%	9.5%
		% of Total	9.5%	0.0%	9.5%
Total		Count	49	14	63
		% within PLRo52	100.0%	100.0%	100.0%
		% of Total	77.8%	22.2%	100.0%

Chi-Square Tests

	Value	df	Asymp. Sig. (2-sided)	Exact Sig. (2-sided)	Exact Sig. (1-sided)
Pearson Chi-Square	1.895[a]	1	.169		
Continuity Correction[b]	.740	1	.390		
Likelihood Ratio	3.192	1	.074		
Fisher's Exact Test				.323	.206
N of Valid Cases	63				

a. 2 cells (50.0%) have expected count less than 5. The minimum expected count is 1.33.

b. Computed only for a 2x2 table

Skin ulcers * PLMi2

Crosstab

			PLMi2		Total
			No	Yes	
Skin ulcers	No	Count	49	8	57
		% within PLMi2	90.7%	88.9%	90.5%
		% of Total	77.8%	12.7%	90.5%
	Yes	Count	5	1	6
		% within PLMi2	9.3%	11.1%	9.5%
		% of Total	7.9%	1.6%	9.5%
Total		Count	54	9	63
		% within PLMi2	100.0%	100.0%	100.0%
		% of Total	85.7%	14.3%	100.0%

Chi-Square Tests

	Value	df	Asymp. Sig. (2-sided)	Exact Sig. (2-sided)	Exact Sig. (1-sided)
Pearson Chi-Square	.031[a]	1	.861		
Continuity Correction[b]	.000	1	1.000		
Likelihood Ratio	.030	1	.864		
Fisher's Exact Test				1.000	.620
N of Valid Cases	63				

a. 1 cells (25.0%) have expected count less than 5. The minimum expected count is .86.

b. Computed only for a 2x2 table

Skin ulcers * PLMDA5

Crosstab

			PLMDA5		Total
			No	Yes	
Skin ulcers	No	Count	49	8	57
		% within PLMDA5	90.7%	88.9%	90.5%
		% of Total	77.8%	12.7%	90.5%
	Yes	Count	5	1	6
		% within PLMDA5	9.3%	11.1%	9.5%
		% of Total	7.9%	1.6%	9.5%
Total		Count	54	9	63
		% within PLMDA5	100.0%	100.0%	100.0%
		% of Total	85.7%	14.3%	100.0%

Chi-Square Tests

	Value	df	Asymp. Sig. (2-sided)	Exact Sig. (2-sided)	Exact Sig. (1-sided)
Pearson Chi-Square	.031[a]	1	.861		
Continuity Correction[b]	.000	1	1.000		
Likelihood Ratio	.030	1	.864		
Fisher's Exact Test				1.000	.620
N of Valid Cases	63				

a. 1 cells (25.0%) have expected count less than 5. The minimum expected count is .86.

b. Computed only for a 2x2 table

Exertional dyspnea * PLantisynthetase

Crosstab

			PLantisynthetase		Total
			No	Yes	
Exertional dyspnea	No	Count	41	11	52
		% within PLantisynthetase	78.8%	100.0%	82.5%
		% of Total	65.1%	17.5%	82.5%
	Yes	Count	11	0	11
		% within PLantisynthetase	21.2%	0.0%	17.5%
		% of Total	17.5%	0.0%	17.5%
Total		Count	52	11	63
		% within PLantisynthetase	100.0%	100.0%	100.0%
		% of Total	82.5%	17.5%	100.0%

Chi-Square Tests

	Value	df	Asymp. Sig. (2-sided)	Exact Sig. (2-sided)	Exact Sig. (1-sided)
Pearson Chi-Square	2.819[a]	1	.093		
Continuity Correction[b]	1.542	1	.214		
Likelihood Ratio	4.689	1	.030		
Fisher's Exact Test				.187	.098
N of Valid Cases	63				

a. 1 cells (25.0%) have expected count less than 5. The minimum expected count is 1.92.

b. Computed only for a 2x2 table

Exertional dyspnea * PLTIF1y

Crosstab

			PLTIF1y		Total
			No	Yes	
Exertional dyspnea	No	Count	38	14	52
		% within PLTIF1y	84.4%	77.8%	82.5%
		% of Total	60.3%	22.2%	82.5%
	Yes	Count	7	4	11
		% within PLTIF1y	15.6%	22.2%	17.5%
		% of Total	11.1%	6.3%	17.5%
Total		Count	45	18	63
		% within PLTIF1y	100.0%	100.0%	100.0%
		% of Total	71.4%	28.6%	100.0%

Chi-Square Tests

	Value	df	Asymp. Sig. (2-sided)	Exact Sig. (2-sided)	Exact Sig. (1-sided)
Pearson Chi-Square	.397[a]	1	.529		
Continuity Correction[b]	.069	1	.793		
Likelihood Ratio	.382	1	.536		
Fisher's Exact Test				.714	.384
N of Valid Cases	63				

a. 1 cells (25.0%) have expected count less than 5. The minimum expected count is 3.14.

b. Computed only for a 2x2 table

Exertional dyspnea * PLRo52

Crosstab

			PLRo52		Total
			No	Yes	
Exertional dyspnea	No	Count	42	10	52
		% within PLRo52	85.7%	71.4%	82.5%
		% of Total	66.7%	15.9%	82.5%
	Yes	Count	7	4	11
		% within PLRo52	14.3%	28.6%	17.5%
		% of Total	11.1%	6.3%	17.5%
Total		Count	49	14	63
		% within PLRo52	100.0%	100.0%	100.0%
		% of Total	77.8%	22.2%	100.0%

Chi-Square Tests

	Value	df	Asymp. Sig. (2-sided)	Exact Sig. (2-sided)	Exact Sig. (1-sided)
Pearson Chi-Square	1.542[a]	1	.214		
Continuity Correction[b]	.710	1	.399		
Likelihood Ratio	1.409	1	.235		
Fisher's Exact Test				.243	.196
N of Valid Cases	63				

a. 1 cells (25.0%) have expected count less than 5. The minimum expected count is 2.44.

b. Computed only for a 2x2 table

Exertional dyspnea * PLMi2

Crosstab

			PLMi2		Total
			No	Yes	
Exertional dyspnea	No	Count	46	6	52
		% within PLMi2	85.2%	66.7%	82.5%
		% of Total	73.0%	9.5%	82.5%
	Yes	Count	8	3	11
		% within PLMi2	14.8%	33.3%	17.5%
		% of Total	12.7%	4.8%	17.5%
Total		Count	54	9	63
		% within PLMi2	100.0%	100.0%	100.0%
		% of Total	85.7%	14.3%	100.0%

Chi-Square Tests

	Value	df	Asymp. Sig. (2-sided)	Exact Sig. (2-sided)	Exact Sig. (1-sided)
Pearson Chi-Square	1.836[a]	1	.175		
Continuity Correction[b]	.776	1	.378		
Likelihood Ratio	1.590	1	.207		
Fisher's Exact Test				.184	.184
N of Valid Cases	63				

a. 1 cells (25.0%) have expected count less than 5. The minimum expected count is 1.57.

b. Computed only for a 2x2 table

Exertional dyspnea * PLMDA5

Crosstab

			PLMDA5		Total
			No	Yes	
Exertional dyspnea	No	Count	46	6	52
		% within PLMDA5	85.2%	66.7%	82.5%
		% of Total	73.0%	9.5%	82.5%
	Yes	Count	8	3	11
		% within PLMDA5	14.8%	33.3%	17.5%
		% of Total	12.7%	4.8%	17.5%
Total		Count	54	9	63
		% within PLMDA5	100.0%	100.0%	100.0%
		% of Total	85.7%	14.3%	100.0%

Chi-Square Tests

	Value	df	Asymp. Sig. (2-sided)	Exact Sig. (2-sided)	Exact Sig. (1-sided)
Pearson Chi-Square	1.836[a]	1	.175		
Continuity Correction[b]	.776	1	.378		
Likelihood Ratio	1.590	1	.207		
Fisher's Exact Test				.184	.184
N of Valid Cases	63				

a. 1 cells (25.0%) have expected count less than 5. The minimum expected count is 1.57.

b. Computed only for a 2x2 table

Fever * PLantisynthetase

Crosstab

			PLantisynthetase		Total
			No	Yes	
Fever	No	Count	49	5	54
		% within PLantisynthetase	94.2%	45.5%	85.7%
		% of Total	77.8%	7.9%	85.7%
	Yes	Count	3	6	9
		% within PLantisynthetase	5.8%	54.5%	14.3%
		% of Total	4.8%	9.5%	14.3%
Total		Count	52	11	63
		% within PLantisynthetase	100.0%	100.0%	100.0%
		% of Total	82.5%	17.5%	100.0%

Chi-Square Tests

	Value	df	Asymp. Sig. (2-sided)	Exact Sig. (2-sided)	Exact Sig. (1-sided)
Pearson Chi-Square	17.641[a]	1	.000		
Continuity Correction[b]	13.882	1	.000		
Likelihood Ratio	13.577	1	.000		
Fisher's Exact Test				.000	.000
N of Valid Cases	63				

a. 1 cells (25.0%) have expected count less than 5. The minimum expected count is 1.57.

b. Computed only for a 2x2 table

Fever * PLTIF1y

Crosstab

			PLTIF1y		Total
			No	Yes	
Fever	No	Count	38	16	54
		% within PLTIF1y	84.4%	88.9%	85.7%
		% of Total	60.3%	25.4%	85.7%
	Yes	Count	7	2	9
		% within PLTIF1y	15.6%	11.1%	14.3%
		% of Total	11.1%	3.2%	14.3%
Total		Count	45	18	63
		% within PLTIF1y	100.0%	100.0%	100.0%
		% of Total	71.4%	28.6%	100.0%

Chi-Square Tests

	Value	df	Asymp. Sig. (2-sided)	Exact Sig. (2-sided)	Exact Sig. (1-sided)
Pearson Chi-Square	.207[a]	1	.649		
Continuity Correction[b]	.003	1	.955		
Likelihood Ratio	.216	1	.642		
Fisher's Exact Test				1.000	.495
N of Valid Cases	63				

a. 1 cells (25.0%) have expected count less than 5. The minimum expected count is 2.57.

b. Computed only for a 2x2 table

Fever * PLRo52

Crosstab

			PLRo52		Total
			No	Yes	
Fever	No	Count	42	12	54
		% within PLRo52	85.7%	85.7%	85.7%
		% of Total	66.7%	19.0%	85.7%
	Yes	Count	7	2	9
		% within PLRo52	14.3%	14.3%	14.3%
		% of Total	11.1%	3.2%	14.3%
Total		Count	49	14	63
		% within PLRo52	100.0%	100.0%	100.0%
		% of Total	77.8%	22.2%	100.0%

Chi-Square Tests

	Value	df	Asymp. Sig. (2-sided)	Exact Sig. (2-sided)	Exact Sig. (1-sided)
Pearson Chi-Square	.000[a]	1	1.000		
Continuity Correction[b]	.000	1	1.000		
Likelihood Ratio	.000	1	1.000		
Fisher's Exact Test				1.000	.684
N of Valid Cases	63				

a. 1 cells (25.0%) have expected count less than 5. The minimum expected count is 2.00.

b. Computed only for a 2x2 table

Fever * PLMi2

Crosstab

			PLMi2		
			No	Yes	Total
Fever	No	Count	46	8	54
		% within PLMi2	85.2%	88.9%	85.7%
		% of Total	73.0%	12.7%	85.7%
	Yes	Count	8	1	9
		% within PLMi2	14.8%	11.1%	14.3%
		% of Total	12.7%	1.6%	14.3%
Total		Count	54	9	63
		% within PLMi2	100.0%	100.0%	100.0%
		% of Total	85.7%	14.3%	100.0%

Chi-Square Tests

	Value	df	Asymp. Sig. (2-sided)	Exact Sig. (2-sided)	Exact Sig. (1-sided)
Pearson Chi-Square	.086[a]	1	.769		
Continuity Correction[b]	.000	1	1.000		
Likelihood Ratio	.091	1	.762		
Fisher's Exact Test				1.000	.620
N of Valid Cases	63				

a. 1 cells (25.0%) have expected count less than 5. The minimum expected count is 1.29.

b. Computed only for a 2x2 table

Fever * PLMDA5

Crosstab

			PLMDA5		Total
			No	Yes	
Fever	No	Count	46	8	54
		% within PLMDA5	85.2%	88.9%	85.7%
		% of Total	73.0%	12.7%	85.7%
	Yes	Count	8	1	9
		% within PLMDA5	14.8%	11.1%	14.3%
		% of Total	12.7%	1.6%	14.3%
Total		Count	54	9	63
		% within PLMDA5	100.0%	100.0%	100.0%
		% of Total	85.7%	14.3%	100.0%

Chi-Square Tests

	Value	df	Asymp. Sig. (2-sided)	Exact Sig. (2-sided)	Exact Sig. (1-sided)
Pearson Chi-Square	.086[a]	1	.769		
Continuity Correction[b]	.000	1	1.000		
Likelihood Ratio	.091	1	.762		
Fisher's Exact Test				1.000	.620
N of Valid Cases	63				

a. 1 cells (25.0%) have expected count less than 5. The minimum expected count is 1.29.

b. Computed only for a 2x2 table

Arthralgia * PLantisynthetase

Crosstab

			PLantisynthetase		Total
			No	Yes	
Arthralgia	No	Count	43	4	47
		% within PLantisynthetase	82.7%	36.4%	74.6%
		% of Total	68.3%	6.3%	74.6%
	Yes	Count	9	7	16
		% within PLantisynthetase	17.3%	63.6%	25.4%
		% of Total	14.3%	11.1%	25.4%
Total		Count	52	11	63
		% within PLantisynthetase	100.0%	100.0%	100.0%
		% of Total	82.5%	17.5%	100.0%

Chi-Square Tests

	Value	df	Asymp. Sig. (2-sided)	Exact Sig. (2-sided)	Exact Sig. (1-sided)
Pearson Chi-Square	10.285[a]	1	.001		
Continuity Correction[b]	7.985	1	.005		
Likelihood Ratio	9.062	1	.003		
Fisher's Exact Test				.004	.004
N of Valid Cases	63				

a. 1 cells (25.0%) have expected count less than 5. The minimum expected count is 2.79.

b. Computed only for a 2x2 table

Arthralgia * PLTIF1y

Crosstab

			PLTIF1y		Total
			No	Yes	
Arthralgia	No	Count	33	14	47
		% within PLTIF1y	73.3%	77.8%	74.6%
		% of Total	52.4%	22.2%	74.6%
	Yes	Count	12	4	16
		% within PLTIF1y	26.7%	22.2%	25.4%
		% of Total	19.0%	6.3%	25.4%
Total		Count	45	18	63
		% within PLTIF1y	100.0%	100.0%	100.0%
		% of Total	71.4%	28.6%	100.0%

Chi-Square Tests

	Value	df	Asymp. Sig. (2-sided)	Exact Sig. (2-sided)	Exact Sig. (1-sided)
Pearson Chi-Square	.134[a]	1	.714		
Continuity Correction[b]	.002	1	.963		
Likelihood Ratio	.136	1	.712		
Fisher's Exact Test				1.000	.491
N of Valid Cases	63				

a. 1 cells (25.0%) have expected count less than 5. The minimum expected count is 4.57.

b. Computed only for a 2x2 table

Arthralgia * PLRo52

Crosstab

			PLRo52		Total
			No	Yes	
Arthralgia	No	Count	36	11	47
		% within PLRo52	73.5%	78.6%	74.6%
		% of Total	57.1%	17.5%	74.6%
	Yes	Count	13	3	16
		% within PLRo52	26.5%	21.4%	25.4%
		% of Total	20.6%	4.8%	25.4%
Total		Count	49	14	63
		% within PLRo52	100.0%	100.0%	100.0%
		% of Total	77.8%	22.2%	100.0%

Chi-Square Tests

	Value	df	Asymp. Sig. (2-sided)	Exact Sig. (2-sided)	Exact Sig. (1-sided)
Pearson Chi-Square	.150[a]	1	.699		
Continuity Correction[b]	.001	1	.969		
Likelihood Ratio	.154	1	.695		
Fisher's Exact Test				1.000	.498
N of Valid Cases	63				

a. 1 cells (25.0%) have expected count less than 5. The minimum expected count is 3.56.

b. Computed only for a 2x2 table

Arthralgia * PLMi2

Crosstab

			PLMi2		Total
			No	Yes	
Arthralgia	No	Count	40	7	47
		% within PLMi2	74.1%	77.8%	74.6%
		% of Total	63.5%	11.1%	74.6%
	Yes	Count	14	2	16
		% within PLMi2	25.9%	22.2%	25.4%
		% of Total	22.2%	3.2%	25.4%
Total		Count	54	9	63
		% within PLMi2	100.0%	100.0%	100.0%
		% of Total	85.7%	14.3%	100.0%

Chi-Square Tests

	Value	df	Asymp. Sig. (2-sided)	Exact Sig. (2-sided)	Exact Sig. (1-sided)
Pearson Chi-Square	.056[a]	1	.813		
Continuity Correction[b]	.000	1	1.000		
Likelihood Ratio	.057	1	.811		
Fisher's Exact Test				1.000	.589
N of Valid Cases	63				

a. 1 cells (25.0%) have expected count less than 5. The minimum expected count is 2.29.

b. Computed only for a 2x2 table

Arthralgia * PLMDA5

Crosstab

			PLMDA5		Total
			No	Yes	
Arthralgia	No	Count	42	5	47
		% within PLMDA5	77.8%	55.6%	74.6%
		% of Total	66.7%	7.9%	74.6%
	Yes	Count	12	4	16
		% within PLMDA5	22.2%	44.4%	25.4%
		% of Total	19.0%	6.3%	25.4%
Total		Count	54	9	63
		% within PLMDA5	100.0%	100.0%	100.0%
		% of Total	85.7%	14.3%	100.0%

Chi-Square Tests

	Value	df	Asymp. Sig. (2-sided)	Exact Sig. (2-sided)	Exact Sig. (1-sided)
Pearson Chi-Square	2.011[a]	1	.156		
Continuity Correction[b]	1.009	1	.315		
Likelihood Ratio	1.825	1	.177		
Fisher's Exact Test				.214	.157
N of Valid Cases	63				

a. 1 cells (25.0%) have expected count less than 5. The minimum expected count is 2.29.

b. Computed only for a 2x2 table

PLClinicalmuscledamge * PLantisynthetase

Crosstab

			PLantisynthetase		Total
			No	Yes	
PLClinicalmuscledamge	No	Count	9	1	10
		% within PLantisynthetase	17.3%	9.1%	15.9%
		% of Total	14.3%	1.6%	15.9%
	Yes	Count	43	10	53
		% within PLantisynthetase	82.7%	90.9%	84.1%
		% of Total	68.3%	15.9%	84.1%
Total		Count	52	11	63
		% within PLantisynthetase	100.0%	100.0%	100.0%
		% of Total	82.5%	17.5%	100.0%

Chi-Square Tests

	Value	df	Asymp. Sig. (2-sided)	Exact Sig. (2-sided)	Exact Sig. (1-sided)
Pearson Chi-Square	.459[a]	1	.498		
Continuity Correction[b]	.050	1	.823		
Likelihood Ratio	.514	1	.473		
Fisher's Exact Test				.676	.440
Linear-by-Linear Association	.452	1	.501		
N of Valid Cases	63				

a. 1 cells (25.0%) have expected count less than 5. The minimum expected count is 1.75.

b. Computed only for a 2x2 table

PLClinicalmuscledamge * PLTIF1y

Crosstab

			PLTIF1y		Total
			No	Yes	
PLClinicalmuscledamge	No	Count	6	4	10
		% within PLTIF1y	13.3%	22.2%	15.9%
		% of Total	9.5%	6.3%	15.9%
	Yes	Count	39	14	53
		% within PLTIF1y	86.7%	77.8%	84.1%
		% of Total	61.9%	22.2%	84.1%
Total		Count	45	18	63
		% within PLTIF1y	100.0%	100.0%	100.0%
		% of Total	71.4%	28.6%	100.0%

Chi-Square Tests

	Value	df	Asymp. Sig. (2-sided)	Exact Sig. (2-sided)	Exact Sig. (1-sided)
Pearson Chi-Square	.761[a]	1	.383		
Continuity Correction[b]	.241	1	.624		
Likelihood Ratio	.722	1	.395		
Fisher's Exact Test				.452	.302
Linear-by-Linear Association	.749	1	.387		
N of Valid Cases	63				

a. 1 cells (25.0%) have expected count less than 5. The minimum expected count is 2.86.

b. Computed only for a 2x2 table

PLClinicalmuscledamge * PLRo52

Crosstab

			PLRo52		
			No	Yes	Total
PLClinicalmuscledamge	No	Count	7	3	10
		% within PLRo52	14.3%	21.4%	15.9%
		% of Total	11.1%	4.8%	15.9%
	Yes	Count	42	11	53
		% within PLRo52	85.7%	78.6%	84.1%
		% of Total	66.7%	17.5%	84.1%
Total		Count	49	14	63
		% within PLRo52	100.0%	100.0%	100.0%
		% of Total	77.8%	22.2%	100.0%

Chi-Square Tests

	Value	df	Asymp. Sig. (2-sided)	Exact Sig. (2-sided)	Exact Sig. (1-sided)
Pearson Chi-Square	.416[a]	1	.519		
Continuity Correction[b]	.053	1	.818		
Likelihood Ratio	.393	1	.531		
Fisher's Exact Test				.679	.390
Linear-by-Linear Association	.409	1	.522		
N of Valid Cases	63				

a. 1 cells (25.0%) have expected count less than 5. The minimum expected count is 2.22.

b. Computed only for a 2x2 table

PLClinicalmuscledamge * PLMi2

Crosstab

			PLMi2		Total
			No	Yes	
PLClinicalmuscledamge	No	Count	10	0	10
		% within PLMi2	18.5%	0.0%	15.9%
		% of Total	15.9%	0.0%	15.9%
	Yes	Count	44	9	53
		% within PLMi2	81.5%	100.0%	84.1%
		% of Total	69.8%	14.3%	84.1%
Total		Count	54	9	63
		% within PLMi2	100.0%	100.0%	100.0%
		% of Total	85.7%	14.3%	100.0%

Chi-Square Tests

	Value	df	Asymp. Sig. (2-sided)	Exact Sig. (2-sided)	Exact Sig. (1-sided)
Pearson Chi-Square	1.981[a]	1	.159		
Continuity Correction[b]	.837	1	.360		
Likelihood Ratio	3.382	1	.066		
Fisher's Exact Test				.332	.187
Linear-by-Linear Association	1.950	1	.163		
N of Valid Cases	63				

a. 1 cells (25.0%) have expected count less than 5. The minimum expected count is 1.43.

b. Computed only for a 2x2 table

PLClinicalmuscledamge * PLMDA5

Crosstab

			PLMDA5		Total
			No	Yes	
PLClinicalmuscledamge	No	Count	10	0	10
		% within PLMDA5	18.5%	0.0%	15.9%
		% of Total	15.9%	0.0%	15.9%
	Yes	Count	44	9	53
		% within PLMDA5	81.5%	100.0%	84.1%
		% of Total	69.8%	14.3%	84.1%
Total		Count	54	9	63
		% within PLMDA5	100.0%	100.0%	100.0%
		% of Total	85.7%	14.3%	100.0%

Chi-Square Tests

	Value	df	Asymp. Sig. (2-sided)	Exact Sig. (2-sided)	Exact Sig. (1-sided)
Pearson Chi-Square	1.981[a]	1	.159		
Continuity Correction[b]	.837	1	.360		
Likelihood Ratio	3.382	1	.066		
Fisher's Exact Test				.332	.187
Linear-by-Linear Association	1.950	1	.163		
N of Valid Cases	63				

a. 1 cells (25.0%) have expected count less than 5. The minimum expected count is 1.43.

b. Computed only for a 2x2 table

ElevatedCK * PLantisynthetase

Crosstab

			PLantisynthetase		Total
			No	Yes	
ElevatedCK	No	Count	31	6	37
		% within PLantisynthetase	59.6%	54.5%	58.7%
		% of Total	49.2%	9.5%	58.7%
	Yes	Count	21	5	26
		% within PLantisynthetase	40.4%	45.5%	41.3%
		% of Total	33.3%	7.9%	41.3%
Total		Count	52	11	63
		% within PLantisynthetase	100.0%	100.0%	100.0%
		% of Total	82.5%	17.5%	100.0%

Chi-Square Tests

	Value	df	Asymp. Sig. (2-sided)	Exact Sig. (2-sided)	Exact Sig. (1-sided)
Pearson Chi-Square	.096[a]	1	.756		
Continuity Correction[b]	.000	1	1.000		
Likelihood Ratio	.096	1	.757		
Fisher's Exact Test				.750	.505
Linear-by-Linear Association	.095	1	.758		
N of Valid Cases	63				

a. 1 cells (25.0%) have expected count less than 5. The minimum expected count is 4.54.

b. Computed only for a 2x2 table

ElevatedCK * PLTIF1y

Crosstab

			PLTIF1y		Total
			No	Yes	
ElevatedCK	No	Count	28	9	37
		% within PLTIF1y	62.2%	50.0%	58.7%
		% of Total	44.4%	14.3%	58.7%
	Yes	Count	17	9	26
		% within PLTIF1y	37.8%	50.0%	41.3%
		% of Total	27.0%	14.3%	41.3%
Total		Count	45	18	63
		% within PLTIF1y	100.0%	100.0%	100.0%
		% of Total	71.4%	28.6%	100.0%

Chi-Square Tests

	Value	df	Asymp. Sig. (2-sided)	Exact Sig. (2-sided)	Exact Sig. (1-sided)
Pearson Chi-Square	.792[a]	1	.373		
Continuity Correction[b]	.368	1	.544		
Likelihood Ratio	.786	1	.375		
Fisher's Exact Test				.408	.271
Linear-by-Linear Association	.780	1	.377		
N of Valid Cases	63				

a. 0 cells (.0%) have expected count less than 5. The minimum expected count is 7.43.

b. Computed only for a 2x2 table

ElevatedCK * PLRo52

Crosstab

			PLRo52		Total
			No	Yes	
ElevatedCK	No	Count	30	7	37
		% within PLRo52	61.2%	50.0%	58.7%
		% of Total	47.6%	11.1%	58.7%
	Yes	Count	19	7	26
		% within PLRo52	38.8%	50.0%	41.3%
		% of Total	30.2%	11.1%	41.3%
Total		Count	49	14	63
		% within PLRo52	100.0%	100.0%	100.0%
		% of Total	77.8%	22.2%	100.0%

Chi-Square Tests

	Value	df	Asymp. Sig. (2-sided)	Exact Sig. (2-sided)	Exact Sig. (1-sided)
Pearson Chi-Square	.566[a]	1	.452		
Continuity Correction[b]	.198	1	.657		
Likelihood Ratio	.560	1	.454		
Fisher's Exact Test				.543	.326
Linear-by-Linear Association	.557	1	.455		
N of Valid Cases	63				

a. 0 cells (.0%) have expected count less than 5. The minimum expected count is 5.78.

b. Computed only for a 2x2 table

ElevatedCK * PLMi2

Crosstab

			PLMi2		
			No	Yes	Total
ElevatedCK	No	Count	31	6	37
		% within PLMi2	57.4%	66.7%	58.7%
		% of Total	49.2%	9.5%	58.7%
	Yes	Count	23	3	26
		% within PLMi2	42.6%	33.3%	41.3%
		% of Total	36.5%	4.8%	41.3%
Total		Count	54	9	63
		% within PLMi2	100.0%	100.0%	100.0%
		% of Total	85.7%	14.3%	100.0%

Chi-Square Tests

	Value	df	Asymp. Sig. (2-sided)	Exact Sig. (2-sided)	Exact Sig. (1-sided)
Pearson Chi-Square	.273[a]	1	.601		
Continuity Correction[b]	.025	1	.875		
Likelihood Ratio	.278	1	.598		
Fisher's Exact Test				.725	.444
Linear-by-Linear Association	.269	1	.604		
N of Valid Cases	63				

a. 1 cells (25.0%) have expected count less than 5. The minimum expected count is 3.71.

b. Computed only for a 2x2 table

ElevatedCK * PLMDA5

Crosstab

			PLMDA5		Total
			No	Yes	
ElevatedCK	No	Count	31	6	37
		% within PLMDA5	57.4%	66.7%	58.7%
		% of Total	49.2%	9.5%	58.7%
	Yes	Count	23	3	26
		% within PLMDA5	42.6%	33.3%	41.3%
		% of Total	36.5%	4.8%	41.3%
Total		Count	54	9	63
		% within PLMDA5	100.0%	100.0%	100.0%
		% of Total	85.7%	14.3%	100.0%

Chi-Square Tests

	Value	df	Asymp. Sig. (2-sided)	Exact Sig. (2-sided)	Exact Sig. (1-sided)
Pearson Chi-Square	.273[a]	1	.601		
Continuity Correction[b]	.025	1	.875		
Likelihood Ratio	.278	1	.598		
Fisher's Exact Test				.725	.444
Linear-by-Linear Association	.269	1	.604		
N of Valid Cases	63				

a. 1 cells (25.0%) have expected count less than 5. The minimum expected count is 3.71.

b. Computed only for a 2x2 table

```
 RECODE Malignancy ('1'=1) (ELSE=0) INTO PLmalignancy.
EXECUTE.
CROSSTABS
  /TABLES=PLmalignancy BY PLTIF1y
  /FORMAT=AVALUE TABLES
  /STATISTICS=CHISQ
  /CELLS=COUNT COLUMN TOTAL
  /COUNT ROUND CELL.
```

Crosstabs

[DataSet1] C:\Users\ADMIN\Desktop\Datadermatomyositis.sav

Case Processing Summary

	Cases					
	Valid		Missing		Total	
	N	Percent	N	Percent	N	Percent
PLmalignancy * PLTIF1y	63	100.0%	0	0.0%	63	100.0%

PLmalignancy * PLTIF1y Crosstabulation

			PLTIF1y		Total
			No	Yes	
PLmalignancy	No	Count	45	17	62
		% within PLTIF1y	100.0%	94.4%	98.4%
		% of Total	71.4%	27.0%	98.4%
	Yes	Count	0	1	1
		% within PLTIF1y	0.0%	5.6%	1.6%
		% of Total	0.0%	1.6%	1.6%
Total		Count	45	18	63
		% within PLTIF1y	100.0%	100.0%	100.0%
		% of Total	71.4%	28.6%	100.0%

Chi-Square Tests

	Value	df	Asymp. Sig. (2-sided)	Exact Sig. (2-sided)	Exact Sig. (1-sided)
Pearson Chi-Square	2.540[a]	1	.111		
Continuity Correction[b]	.229	1	.633		
Likelihood Ratio	2.546	1	.111		
Fisher's Exact Test				.286	.286
Linear-by-Linear Association	2.500	1	.114		
N of Valid Cases	63				

a. 2 cells (50.0%) have expected count less than 5. The minimum expected count is .29.

b. Computed only for a 2x2 table
