# Supplementary material for: The association between clinical, subclinical features and autoantibody profile in Vietnamese dermatomyositis patients
Source: PLOS Glob Public Health. 2023 Jan 12;3(1):e0000979. doi: 10.1371/journal.pgph.0000979 (PMC10021228; doi:10.1371/journal.pgph.0000979)
Supplement: Attachment — Submitted filename: Response to Reviewers.docx [file pgph.0000979.s003.docx]

# Thank you for considering our manuscript. We appreciate the reviewers’ comments and have incorporated much of their feedback into the manuscript.

# **Reviewer #1:**

# 1. The title is vague because the comparison is not clear; is clinical and subclinical feature compared with autoantibody profile? for me, the comparison seems among the three (clinical, subclinical, and autoantibody profiles), and the clinical and subclinical features are not stated separately.

# *Thank you for your comments. However, regarding the title, our study did not compare the clinical and laboratory characteristics of patients with antibody profile testing. This study was conducted with the aim of investigating the relationship between autoantibody test and clinical and subclinical characteristics of patients with dermatomyositis. From these results, we hope that based on antibody test results, we can predict the possibility of clinical and subclinical symtomps. Therefore, I think this title perfectly fits the purpose of our study.*

# 2. The study participants, according to the title, are dermatomyositis patients but in the methodology part and in the result the study participants are diabetic patients (72 DM patients). and again the patients with different diagnoses (dermatitis and myositis) are included in the title. I don't, do the authors mean patients with both manifestations? if that is so, in the metrology the study participants should be patients having both dermatitis and myositis.

# *Thank you for this feedback. However, we think that there was a misunderstanding. Our study objects were dermatomyositis patient, or in abbreviation DM, in stead of diabetes. We apologize for causing unnecessary misunderstanding, and we have edited the manuscript.*

# *In the diagnostic criteria of dermatomyositis, clinical skin lesions are required while muscle lesions can be observed clinically or detected by laboratory tests.*

# *We would like to emphasize that all patients included in this study had a definitive diagnosis of dermatomyositis according to the diagnostic criteria of Tanimoto et al. 1995. Therefore, the study population only included patients with dermatomyositis as in the title, no patients with dermatitis and myositis were involved.*

# 3. Almost all parts of the introduction talk about DM which is not part of the study. I knew that DM has many complications on different organs. It can be mentioned that one of the causes of autoantibody profile change will be DM, but in this manuscript, DM was mentioned as the study is about DM.

# *Thank you for your comments. We will edit the introduction to highlight the main purpose of the study. The introduction was explain why we conducted this study. Dermatomyositis causes many complications in different organs and is associated with the presence of autoantibodies, which, in turn, may be related to the organ damage mentioned. This study was conducted to investigate the relations of autoantibodies found in dermatomyositis patients and their clinical and laboratory features.*

# 4. The study participants were DM in the methodology but in the title, it is mentioned that the study participants were dermatomyositis.

# *As I explained in your 2nd comment, DM stands for Dermatomyositis. Both refer to one object.*

# 5. The statistical analysis was not clearly written. for Example the soft was for data entry was not mentioned, the presentation of variables like categorical and continuous were not explained well.

# *Our research uses SPSS 20.0 software for both data entry and data analysis. As you mentioned, we will edit the Statistical methods section to clarify the statistical tests used in data analysis.*

# 6. The first paragraph, in the result part, is not in line with the title.

# *As you mentioned, we will edit the first part of the study results section to be more concise. However, we believe that the statistics on clinical and subclinical characteristics of the study group as shown in Tables 1,2,3 are essential to* *highlight the specific characteristics of this population - dermatomyositis patients in Vietnam. In addition, it helps to analyze the correlations between clinical, laboratory and antibody tests more comprehensively.*

# 7. The first paragraph of the discussion focused on DM which is not part of the study.

# *As we explained in response 6, we believe that a better understanding of the characteristics of the study population will help better understand the association between clinical and laboratory characteristics and antibody testing in the Vietnamese patient population.*

# 8. The way the authors concluded is not appropriate. the result should be about the association between clinical and subclinical futures and autoantibody profile among dermatomyositis patients, and the conclusion will be drawn from the result. but the conclusion was about DM which should never ever be the result of this study.

# *Thank you for your comments. We will edit the conclusion to better match the results and title of the study.*

# **Reviewer #2:**

# Most of results were interesting to understand and the data is comprehensive and well presented in the tables, and the experimental approaches appear sound. However, the manuscript needs major modifications to be considered for possible publication First of all, text should be reviewed and corrected by native speaker. Authors should redescribe Discussion part. The Discussion should not be repeated on the Results. The results of this study should be compared with those of previous studies and the cause of these differences should be described.

# *Thank you so much for your feedback. We will review and correct the manuscript with the help of a native speaker.* *Besides, we will review and edit the discussion to make it more appropriate. You can view it in the Revised Manuscript.*

# These are our responses. If you need more information or find it inappropriate, please let us know. Thank you very much for your valuable and sincere comments.
