# Supplementary material for: The association between clinical, subclinical features and autoantibody profile in Vietnamese dermatomyositis patients
Source: PLOS Glob Public Health. 2023 Jan 12;3(1):e0000979. doi: 10.1371/journal.pgph.0000979 (PMC10021228; doi:10.1371/journal.pgph.0000979)
Supplement: Attachment — Submitted filename: Response to Reviewers.docx [file pgph.0000979.s004.docx]

***Thank you for considering our manuscript. We appreciate the reviewers’ comments and have incorporated much of their feedback into the manuscript.***

**Reviewer #2:** The authors have tried to respond well to major concerns, but still the manuscript requires some improvement
Introduction: The authors here they have just provided the knowledge gap or statement of the problem. The authors should provide background information on dermatomyositis and auto-antibodies.
Materials and Methods: The authors used The EUROLINE Immunoblot assay and goes ahead and states that “The incubation protocol includes the following steps:” This sections should always be written in continuous prose instead of stating the steps.
Statistical methods: The authors should be more specific how Pearson’s χ2 test or Fisher’s exact test was employed and in respect to which analysis
Conclusion: The conclusion by the authors is vague, they should provide a concrete conclusion without necessary providing statements on the results.

*Thank you very much for your feedback. We have reviewed and revised the manuscript to better accommodate your valuable comments. You can view it in the Revised Manuscript.*

**Reviewer #3: (No Response)**

*Thank you for reviewing and commenting on our manuscript.*

***These are our responses. If you need more information or find it inappropriate, please let us know. Thank you very much for your valuable and sincere comments.***
